# Characterization on the oncogenic effect of the missense mutations of p53 via machine learning

**DOI:** 10.1093/bib/bbad428

**Published:** 2023-11-28

**Authors:** Qisheng Pan, Stephanie Portelli, Thanh Binh Nguyen, David B Ascher

**Affiliations:** School of Chemistry and Molecular Bioscience, University of Queensland, Brisbane Queensland 4072, Australia; Computational Biology and Clinical Informatics, Baker Heart and Diabetes Institute, Melbourne Victoria 3004, Australia; School of Chemistry and Molecular Bioscience, University of Queensland, Brisbane Queensland 4072, Australia; Computational Biology and Clinical Informatics, Baker Heart and Diabetes Institute, Melbourne Victoria 3004, Australia; School of Chemistry and Molecular Bioscience, University of Queensland, Brisbane Queensland 4072, Australia; Computational Biology and Clinical Informatics, Baker Heart and Diabetes Institute, Melbourne Victoria 3004, Australia; School of Chemistry and Molecular Bioscience, University of Queensland, Brisbane Queensland 4072, Australia; Computational Biology and Clinical Informatics, Baker Heart and Diabetes Institute, Melbourne Victoria 3004, Australia

**Keywords:** missense mutation, p53, protein structure, machine learning analysis

## Abstract

Dysfunctions caused by missense mutations in the tumour suppressor p53 have been extensively shown to be a leading driver of many cancers. Unfortunately, it is time-consuming and labour-intensive to experimentally elucidate the effects of all possible missense variants. Recent works presented a comprehensive dataset and machine learning model to predict the functional outcome of mutations in p53. Despite the well-established dataset and precise predictions, this tool was trained on a complicated model with limited predictions on p53 mutations. In this work, we first used computational biophysical tools to investigate the functional consequences of missense mutations in p53, informing a bias of deleterious mutations with destabilizing effects. Combining these insights with experimental assays, we present two interpretable machine learning models leveraging both experimental assays and *in silico* biophysical measurements to accurately predict the functional consequences on p53 and validate their robustness on clinical data. Our final model based on nine features obtained comparable predictive performance with the state-of-the-art p53 specific method and outperformed other generalized, widely used predictors. Interpreting our models revealed that information on residue p53 activity, polar atom distances and changes in p53 stability were instrumental in the decisions, consistent with a bias of the properties of deleterious mutations. Our predictions have been computed for all possible missense mutations in p53, offering clinical diagnostic utility, which is crucial for patient monitoring and the development of personalized cancer treatment.

## INTRODUCTION

Dysfunctions caused by genetic variants on tumour suppressor p53 is one of the leading drivers of many cancers and has attracted considerable attention within the cancer research realm over the past five decades [1]. Of these genetic variants, over 50% are reported in cancer patients with a defective p53 [2–4], and over 90% of these oncogenic mutations are missense mutations [5]. Further to that, germline mutations in p53 also cause Li–Fraumeni syndrome (LFS), predisposing patients to cancer [6].

**Figure 1:**
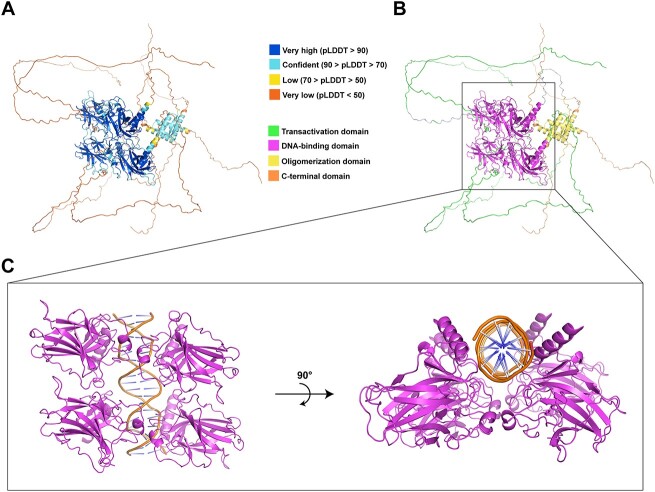
Complete protein structure of tumour suppressor p53 generated by AlphaFold2. Structure is coloured based on the AlphaFold2 confidence score pLDDT in four levels, namely Very high (blue), Confident (cyan), Low (yellow), and Very low (orange) (A), and different domains, namely Transactivation domain (green), DNA-binding domain (magenta), Oligomerization domain (yellow), and C-terminal domain (orange) (B). The DNA-binding domain is zoomed in to show more details (C).

The canonical p53 protein is a homotetramer consisting of 393 residues of each protomer. Four main functional domains in p53 have been well distinguished, namely, transactivation domain (TAD) on the N-terminal, DNA-binding domain (DBD), oligomerization domain (OD) and C-terminal domain (CTD) [7–11] (Figure 1). The structure of both TAD and CTD are usually in an intricate, disordered conformation, but they will fold in a certain conformation when p53 interacts with different protein binding partners [7, 10, 12], while the DBD and OD have specific conformations for DNA binding and tetramerization.

At the cellular level, p53 is responsible for DNA damage–induced activation in the regulation of cell division [13–15], cellular apoptosis [16, 17] and the response of external and internal stress [18, 19]. In the normal unstressed state, a low protein level of p53 is maintained by MDM2, an E3 ubiquitin ligase [20]. Under DNA damage stress, p53 plays a primary role in DNA repair for the erroneous DNA replication by halting the cell cycle, guarding the genome stability [21, 22]. Meanwhile, p53 mediates the expression of genes that encode apoptotic proteins, such as the BCL-2 protein family, by working as a transcription factor [17, 23, 24] to activate the cellular program death before the growth of tumour. Deleterious missense mutations on p53, especially on the DBD, could have a dramatic effect on how p53 folds [25], its stability [26] and the interaction with macromolecules [27] and result in severe functional changes as well as phenotypes [1, 28], which is almost impossible to experimentally elucidate, due to poor correlation with phenotypic data, being time consuming and high cost.

While there are several available methods to evaluate the impact of mutations on p53, these approaches have different limitations. Several predictive models were dependent on experimental assays of high cost, including immunohistochemistry [29], expression data [30, 31] and functional assays [32]. On the other hand, the conventional generic variant effect predictors [33–35] tended to be less accurate in assessing mutation effects on a specific protein. A recent p53-specific study, TP53_PROF [36], combined both experimental signatures and computational scores to develop machine learning models. Despite the robust performance, this method was more complex, utilizing 42 variables, and failed to provide a complete prediction of all possible missense mutations.

Previously our machine learning-based pipeline has been applied on predicting the mutation effects of pathogenicity [37–39] and drug resistance [40–42] across different proteins. In the current work, we adopted a similar supervised machine learning approach to comprehensively map p53 mutation functional outcomes for a better understanding of p53-mediated carcinogenesis. Our final model which integrated experimental assays obtained high performance, and relied on contributions from structure-based computational biophysical measurements. Comparable performance was also obtained using only *in silico* features, making this method experimental assay-agnostic and more accessible, by reducing the dependence on costly experiments. Compared with the state-of-the-art methods, our final model used only nine interpretable features to make accurate predictions and has wider applications as the predictions of all possible missense mutations of p53 have been precalculated. This work therefore has significant clinical and research-based applications, as it offers an understanding of the cancer mechanisms, which can guide the development of novel treatments [43], while also enabling knowledge-driven clinical diagnosis for somatic cancer and LFS.

## MATERIALS AND METHODS

**Figure 2:**
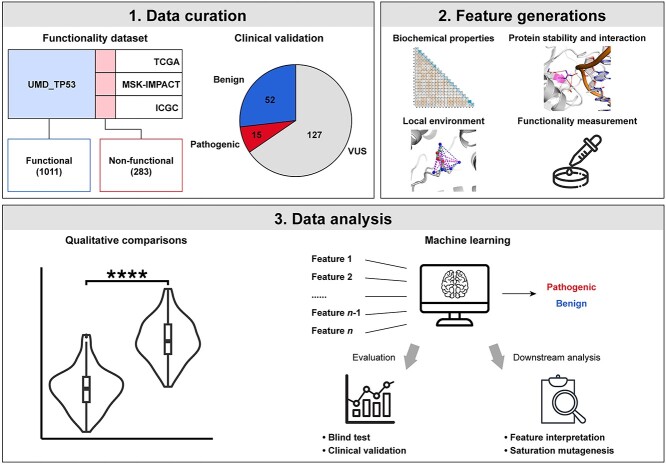
The workflow to characterize oncogenic missense variants of tumour suppressor p53. Two mutation datasets, the functionality dataset and the clinical validation set, were generated by collecting mutations with labels from multiple resources. In the functionality dataset, non-functional missense mutations were the intersection among cancer cases from the Universal Mutation Database TP53 (UMD_TP53) and three other cancerous databases, while the functional mutations were the unique/absent mutations in the UMD_TP53. The whole dataset was curated by another work TP53_PROF. Clinical validation set was established by using the American College of Medical Genetics and Genomics and the Association for Molecular Pathology (ACMG/AMP) guidelines and literature search to confirm clinical diagnosis of mutations in ClinVar to collect pathogenic, benign and variant with unknown significance (VUS). After this, mutations were characterized using various features including biochemical properties, protein stability and interactions, local environment and functionality assessments. Finally, the qualitative comparisons and machine learning analysis were used to identify potential risk factors leading to cancer, followed by feature interpretation and *in silico* saturation mutagenesis.

The overall aim of this work was to accurately characterize oncogenic missense mutations in p53, providing a comprehensive landscape of all possible missense mutations via *in silico* mutagenesis, whose workflow is depicted in Figure 2.

### p53 missense mutation datasets with functional labels

A recently published comprehensive dataset describing a total of 1294 mutations [36] was employed for primary analysis on p53 protein in this work. Briefly, these data were obtained from the Universal Mutation Database (UMD) TP53 database (2019_R1 release) [2, 44], a locus-specific database for the tumour suppressor p53 and three cancer databases: The Cancer Genome Atlas Program (TCGA) [45], MSK-IMPACT Clinical Sequencing Cohort [46] and the International Cancer Genome Consortium (ICGC) data portal [47]. Cancer-related variants at the intersection of all four databases were extracted to construct a non-functional class (*n =* 283), while mutations that failed that criterion were retained as the functional class (*n* = 1011). According to the source of dataset [36], non-functional mutations have strong evidence in human cell experiments that they tend to disrupt p53 function on transcriptional and cell cycle arrest activity, while the functional mutations had mild impacts on the protein. This functionality dataset was used for characterizing pathogenic molecular drivers and initial model development.

### Independent clinical assessment on p53 missense variants

To validate the clinical utility of our model predictions, an independent p53 missense mutation dataset was curated from the ClinVar database [48]. After removing the redundant entries, all the missense mutations were included in the dataset if their phenotypes were validated based on either the American College of Medical Genetics and Genomics and the Association for Molecular Pathology (ACMG/AMP) guidelines [49], or the Clinical Genome Resource TP53 Variant Curation Expert Panel (ClinGen TP53 VCEP) [50], which is a specific interpretation of germline variants on p53. In contrast to the functional impact defined in the functionality dataset, this independent clinical test set was based on more robust criteria of clinical pathogenicity.

After removing the variants with unknown significance (VUS) and the overlapped mutations with the functionality dataset, our final clinical test set (Supplementary File available online at http://bib.oxfordjournals.org/) contained 67 mutations labelled ‘benign/likely benign’ (*n* = 52) and ‘pathogenic/likely pathogenic’ (*n* = 15). Further to that, all the variants labelled as ‘pathogenic/likely pathogenic’ were manually verified through the literature, to ensure that the final mutations tested were observed in clinical patients manifesting disease.

### Structural information of p53 homotetramer

Due to the flexibility of the native structure, no experimental full-length homotetramer was available (as of March 2023) from the RCSB Protein Data Bank (PDB) database. Because of this, we generated the full protein structure using AlphaFold2 v2.1 (AF2) [51]. As the robustness of using AF2 models for computational biophysical measurements has been demonstrated in our previous work [52], we expected that our results can achieve similar findings as using experimental structures.

Tetrameric p53 models (UniProt ID: P04637; canonical form) were built using AlphaFold2-multimer [53] with the genetic databases and template parameters as of March 2022. The model with the highest confidence score, measured by predicted Local Distance Difference Test (pLDDT) [54], was selected. As expected, only the DBD and OD regions showed high structural confidence (pLDDT > 80), and, when compared to their respective crystal structures, the DBD had an all-atom root mean squared deviation (RMSD) of 1.12 Å (PDB ID: 6FJ5 [9]), while the OD had an RMSD of 0.31 Å (PDB ID: 1C26 [55]) (Figure 1).

Atom coordinates of the double-stranded DNA (dsDNA) and Zn^2+^ atom were transferred from the experimental crystal structure (PDB ID: 6FJ5) describing the interaction between p53 DBD and dsDNA to the AF2 model to calculate the effects of mutations on nucleic acid and metal binding, respectively. In doing so, two out of four p53 monomers were observed to have clashes with the transferred dsDNA. As the overall monomer is mostly symmetric [56], changes in nucleic acid affinity were calculated on one chain (B) and assumed representative for all chains. Final tetrameric p53 models are available in the Supplementary Files available online at http://bib.oxfordjournals.org/.

### Biochemical and functional annotations of missense variants

In this work, the effects of missense variants were described from four aspects: biochemical properties, local residue environment, protein stability and interaction and functionality assessment. All the categorical features were transformed using one-hot-encoding.

#### Biochemical properties and changes in conservation

Residue substitutions may impact conserved sequence motifs causing important functional disruptions. To measure these changes, the biochemical properties generated from multiple sequence alignments were extracted from the AAindex 2 and 3 databases [57]. Position-specific substitution matrices (PSSM) were calculated by PSI-BLAST [58, 59], searching against the non-redundant (*nr)* database [60], with three iterations, to study position-specific effects.

The missense tolerance ratio (MTR) was obtained from MTR-viewer [61] and MTR-3D resources [62]. The MTR score highlights regions within a gene under purifying selection, acting as a measure of deleteriousness based on variation observed within a species. The application of this score to 3D protein structures is given by MTR-3D. The MTR scores were obtained based on different sliding windows (21, 31 and 41 codons), while MTR-3D scores were generated for the DBD (using PDB ID: 6FJ5) and OD (using PDB ID: 1C26).

#### Local structural environment

Computational structural information was calculated to account for the local environmental context of each mutation. Local structural environment was established from three perspectives.

##### Basic structural environment

Solvent exposure represented by relative solvent accessibility (RSA) and residue depth were calculated via Biopython [63]. Secondary structure type, phi and psi angles were computed via the DSSP program [64, 65]. In addition, the pLDDT scores from the output of the AF2 structures were also used as a representation of protein disorder.

##### Residue contacts

The local environment of various residue interactions of both wild-type (WT) and mutants (generated by MODELLER [66–68]) was calculated via Arpeggio [69].

##### Graph-based signatures

The atomic distance patterns called graph-based signatures were calculated using mCSM-suite [70]. In the graph-based signatures, atoms with different pharmacophores are considered as nodes and their contacts are considered as edges. By setting different distance cutoffs and distance steps systematically, multiple patterns of atom pairs were captured. In this work, the changes of pharmacophores of eight different types of atoms were calculated for different mutations. The signatures of hydrophobic and polar atom pairs were calculated to model the mutation environment.

#### Protein thermodynamic stability and interaction

The change of protein stability upon mutation were calculated by structure-based programs mCSM-Stability [70], DUET [71], SDM [72], ENCoM [73], DynaMut [74], DynaMut2 [75] and the sequence-based program SAAFEC-SEQ [76]. Results from SAAFEC-SEQ were generated using the *nr* database as suggested. The change of protein–protein interaction (PPI) affinity on different monomer combinations was calculated by mCSM-PPI [70] and mCSM-PPI2 [77]. The change of protein–dsDNA interaction was calculated by mCSM-DNA [70] and mCSM-NA [78]. The results from all the computational tools above measured a change in Gibbs free energy (∆∆G) with zero as cutoff (∆∆G < 0: destabilizing; ∆∆G > 0 stabilizing). The change of energy fluctuation was computed using the normal mode analysis of ENCoM through DynaMut. The distance from the mutation site to the Zn^2+^ atom was also computed to represent metal-binding regions.

Structure-based features were only computed for the mutations in the DBD and OD using the full p53 structure as input, while features of mutations outside were marked as non-affected values because of the disordered conformation. We tried to see whether the features generated from the complete structure could consider the global relationship between DBD and OD.

#### Functionality measurements

In this work, we integrated experimental data generated by a previous deep mutational scanning (DMS) assay on p53 because of its high coverage through saturation mutagenesis [32]. Functional annotation was performed on the A549 human lung carcinoma cell lines through p53-Mutagenesis by Integrated TilEs (MITE) screening [79] with two p53-activating agents, nutlin-3 and etoposide. The enrichment or depletion of each amino acid substitution was measured by high-throughput sequencing to produce a Z-score, which quantified their fitness towards p53 protein. The functionality measurements from these experimental assays sought to find dominant negative, loss-of-function and WT-like variants. We used the raw value of fitness scores available in this experimental assay [32].

Four widely used computational functional predictors, namely, SIFT [35], PolyPhen2 [34], PROVEAN [33] and SNAP2 [80], were also introduced. The functional scores obtained from these tools are based on the alignment of multiple genetic databases to identify deleterious mutations, which could be a reference for our models in the absence of experimental functional measurements.

### Qualitative analysis

To identify potential oncogenic molecular drivers, a two-tailed Wilcoxon signed-rank test was implemented to statistically compare the difference between functional and non-functional mutations of numerous computational biophysical measurements and the basic structural environment. Features could be considered as a potential oncogenic risk factor if the difference reached the significant level (*P*-value < 0.05).

### Supervised machine learning

#### Model development

The p53 functionality dataset was divided into a training set (80%) and a blind test set (20%), and the training set was further divided into 75% (60% of original dataset) sub-training and 25% (20% of original dataset) validation for greedy feature selection. All mutations with the same WT residue positions were grouped and were only split into one of the subsets (training, validation or blind test), to reduce the redundancy and enhance the generalization of the machine learning model. Meanwhile, we also maintained a similar distribution of positive and negative labels in all three subsets. Predictive performance was measured by recall, precision, balanced accuracy (BACC), F1-score, Matthew’s correlation coefficient (MCC) and area under the receiver operating characteristic (ROC) curve (AUC).

The machine learning–based analysis has been well illustrated in previous works [38]. A number of machine learning algorithms (DecisionTrees, RandomForest, ExtraTrees, AdaptiveBoosting, GradientBoosting, ExtremeGradientBoosting, NaiveBayesClassifier, SupportVectorMachine, LogisticRegression, K-NearestNeighbour and Multi-layerPerceptron) were tested and the model with best predictive performance using the *scikit-learn* package (v1.0.2), measured through MCC was further optimized via greedy feature selection, which has been explained in our previous works [75, 81].

#### Feature interpretation

To explore how the *in silico* features perform when the experimental data were absent, we conducted two predictive models with different feature sets. The first feature set called ExpAssay contained all 289 features including the fitness scores from experimental assays, while the other one called noExpAssay solely excluded the experimental measurements. Other data preprocessing remained the same on both these two feature sets so that we could adequately compare the contributions of the computational structural features. Feature importance was interpreted using the SHapley Additive exPlanations (SHAP) value [82].

#### Comparison of predictive performance

Four types of computational approaches, namely, p53-specific, DMS-based, conservation-based and deep learning-based methods, were chosen for performance benchmark. TP53_PROF [36] was the latest p53-specific method using both functional and computational scores to predict missense mutations in p53 protein. DeMaSk [83], Envision [84] and VARITY [85] are three DMS-based methods, giving a quantitative value to measure the effect of missense mutations on proteins. We first used the training set to find an optimized cutoff based on MCC and applied the same cutoff on the blind test for performance benchmark. The final cutoffs of DeMaSk, Envision and VARITY we used were −0.28, 0.67 and 0.93, respectively. The conservation-based methods, including SIFT, PolyPhen2, PROVEAN and SNAP2, were also introduced for comparisons by using their suggested cutoff of 0.05, 0.45, −2.50 and 0.00, respectively. AlphaMissense [86], ESM-1b [87] and CPT-1 [88], the methods built from the sophisticated deep learning architecture and protein language models, were nominated to compare the predictive performance on effect of p53 mutations. All these methods also provided the quantitative measurement of the effect of mutations, and AlphaMissense has the suggested cutoffs (‘likely benign’ if score is less than 0.34, ‘likely pathogenic’ if score is higher than 0.56 and ‘ambiguous’ if score is between 0.34 and 0.56). We applied the same methods of the DMS-based methods to determine a cutoff for these methods to remove ambiguity (AlphaMissense: 0.87, ESM-1b: −9.97, CPT-1: 0.55).

## RESULTS

### Mutation phenotype distribution

**Figure 3:**
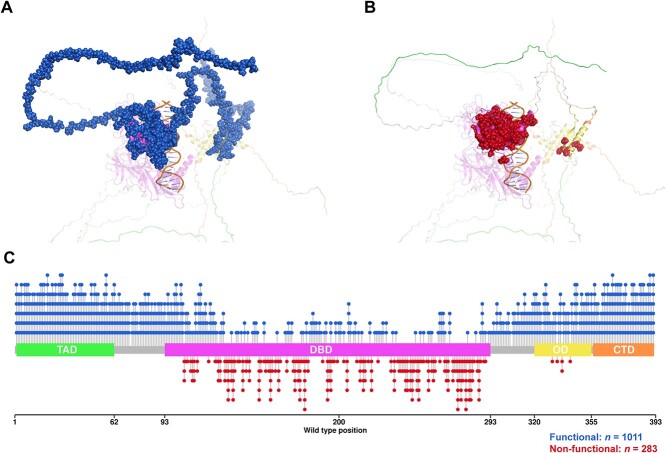
Distribution of the phenotypes caused by missense mutations on protein structure (**A**, **B**) and sequence **(C**) of p53 protein. The red and blue labels represented the non-functional and functional outcomes, respectively. Four main domains of p53, namely, transactivation domain (TAD), DNA-binding domain (DBD), oligomerization domain (OD) and C-terminal domain (CTD), were coloured in green, magenta, yellow and orange, respectively.

The p53 functional mutations were uniformly distributed across the whole sequence (Figure 3), while non-functional mutations clustered mostly in the DBD (*n* = 278) and the OD (*n* = 5). This highlights the functional importance of these domains. Further to that, 47.06% missense mutations are related to the substitution among the hydrophobic (A, F, I, L, M, V, W and Y), polar (N, Q, S and T) and special (C, G and P) residues (Figure S1 available online at http://bib.oxfordjournals.org/). The ‘from special’ and ‘to special’ mutations are observed to have a bias on non-functional phenotypes, consistent with their unique properties in protein structures. In our splitting of the train and blind test, we grouped the mutations based on the WT residue position to reduce redundancy. Distribution of the mutations in the OD was 3:2 in the training and blind test sets, respectively.

### Exploring oncogenic molecular drivers of p53

Potential molecular drivers leading to the development of cancer were proposed using qualitative comparisons across numerous biophysical terms, namely, protein stability, PPI affinity, protein–dsDNA binding affinity and protein–metal distance, as well as the basic structural environment, namely, RSA, residue depth, torsion angles of peptide bonds and the protein disorderedness.

#### Biophysical properties

**Figure 4:**
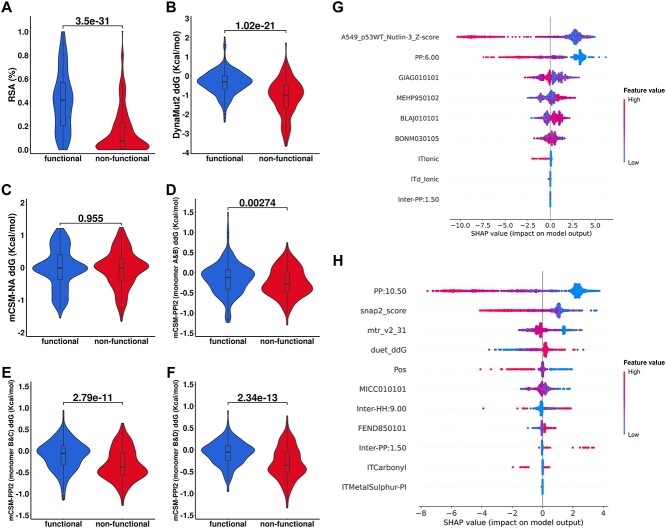
Exploring the potential molecular drivers leading to cancer. Qualitative analysis (Wilcoxon signed-rank test) on the relative solvent accessibility (RSA) at mutation site (**A**), mutation effect on protein stability computed by DynaMut2 (**B**), mutation effect on protein-double-stranded DNA binding affinity computed by mCSM-NA (**C**) and mutation effect on the protein–protein interaction computed by mCSM-PPI2 (**D**–**F**) were applied to compare the functional and non-functional mutations in DBD at 5% significance level. Features were computed on the monomer B of tetrameric p53. The p53 homotetramer is formed as a dimer of dimer. Monomer A and B are at the dimer–dimer interface, while monomer B and C are at the dimer interface. Monomer B and D are at the diagonal position. Feature importance of the ExpAssay (**G**) and noExpAssay (**H**) models was evaluated by SHapley Additive exPlanations (SHAP). Features were ordered from top to bottom based on their contributions to the models. To interpret the plot, the contribution of features towards different labels is represented by the direction of the *x*-axis (negative: non-functional; positive: functional), while the numeric value of feature is represented by the colour of each data point. Combining these two aspects, contributions of each feature could be interpreted.

The investigation of mutation effects by these computational biophysical measurements indicates that destabilizing effects on both monomeric and tetrameric states of mutated p53 are strongly associated with its dysfunctions. Considering stability of the protomer, six of our predictors consistently highlighted that DBD non-functional mutations caused a more significant destabilizing effect, with ΔΔG values as disruptive as −3.5 Kcal/mol (*P*-value = 1.02 × 10^−21^, Figure 4B and Figure S2 available online at http://bib.oxfordjournals.org/). The effect of non-functional mutations on protein dynamics was similar to the effect of the functional ones (Figure S1A available online at http://bib.oxfordjournals.org/). As for the global stability formed by PPI between monomers, we noticed that the non-functional mutations tend to be mildly destabilized on interaction within the dimer and across the dimer–dimer interface, suggested by the statistical comparisons on systematic monomer combinations (*P*-value = 2.34 × 10^−13^–2.74 × 10^−3^, Figure 4D–F and Figure S3 available online at http://bib.oxfordjournals.org/). However, the destabilizing effect caused by non-functional mutations is not shown in the protein–dsDNA complex, despite the closer positions of deleterious mutations (*P*-value = 0.955, Figure 4C and Figure S4 available online at http://bib.oxfordjournals.org/). The non-functional mutations also tend to be located closer to the Zn^2+^ atom (*P*-value = 3.93 × 10^−19^, Figure S5 available online at http://bib.oxfordjournals.org/), which is also relevant to the stability of p53 [89], consistent with our results on protein stability.

#### Structural environment

The descriptions of the structural environment of non-functional mutations agreed with the bias of destabilizing effect found in the biophysical measurements. Deleterious mutations tend to be located in the buried, rigid region of p53, suggested by their solvent accessibility (Figure 4A and Figure S6A and B available online at http://bib.oxfordjournals.org/), which may have a stronger impact on the protein stability. There is a small difference in protein disorder as measured by pLDDT score and the location of mutations of protein backbone (Figure S6C–E available online at http://bib.oxfordjournals.org/).

### Predictive performance of p53 missense mutations towards functionality and cancer

Given the initial distinctions observed across different features, we next adopted a machine learning–based analysis to study the relationship of mutated p53 and functionality and oncogenicity. Two models, ExpAssay and noExpAssay, were developed based on the ExtremeGradientBoosting and GradientBoosting algorithms, respectively. The ExpAssay model achieved an MCC of 0.88 on the blind test (Table 1 and Table S1 available online at http://bib.oxfordjournals.org/), showing high capability of distinguishing classes. Meanwhile, the ExpAssay model shows minimal bias on two phenotypes (F1-score = 0.98, recall = 0.99, precision = 0.96). This is likely due to the high signal-to-noise ratio for functional characterization contributed by the experimental data. However, using solely *in silico* features, we also achieved a comparable performance on the bind test (MCC = 0.81, F1-score = 0.96) (Table 1), implying the high reliability of the computational features. In terms of the performance on the clinical validation, both models display little performance deterioration when compared with the ones in the blind test (ExpAssay MCC = 0.83, noExpAssay MCC = 0.78, Table 2), indicating not only high generalizability of the machine learning models but also potential utility in the clinical environment.

**Table 1:** Predictive performance of different variant effect predictors on the functionality dataset

Models	Method type	Test set^a^	MCC^b^	BACC^b^	F1-score	AUC^b^	Recall	Precision
ExpAssay	p53-specific	10-CV	0.91	0.95	0.98	0.99	0.99	0.98
noExpAssay		10-CV	0.78	0.89	0.95	0.97	0.95	0.95
ExpAssay		Blind test	0.88	0.92	0.98	0.99	0.99	0.96
noExpAssay		Blind test	0.81	0.87	0.96	0.98	0.99	0.94
TP53_PROF		Blind test	0.88	0.94	0.90	0.99	0.91	0.90
DeMask	Deep mutation scanning (DMS)–based	Blind test	0.54	0.78	0.89	0.91	0.88	0.91
Envision		Blind test	0.60	0.77	0.92	0.89	0.95	0.89
VARITY		Blind test	0.72	0.88	0.93	0.95	0.91	0.96
SIFT	Conservation-based	Blind test	0.46	0.78	0.74	0.88	0.59	0.98
PolyPhen2		Blind test	0.42	0.75	0.70	0.87	0.54	0.98
SNAP2		Blind test	0.58	0.88	0.84	0.95	0.72	0.99
PROVEAN		Blind test	0.69	0.89	0.91	0.95	0.86	0.97
AlphaMissense	Deep learning–based	Blind test	0.78	0.89	0.95	0.97	0.95	0.95
ESM-1b		Blind test	0.68	0.83	0.93	0.95	0.94	0.93
CPT-1		Blind test	0.68	0.80	0.94	0.95	0.97	0.91

^a^Stratified group 10-fold cross validation was performed, grouping mutations by WT residue position to reduce redundancy.

^b^MCC stands for Matthew’s correlation coefficient. BACC stands for balanced accuracy. AUC stands for area under the receiver operating characteristic (ROC) curve.

**Table 2:** Predictive performance of the pathogenic outcome of missense mutations from the clinical validation

Models	Method type	MCC	BACC	F1-score	AUC	Recall	Precision
ExpAssay	p53-specific	0.83	0.87	0.96	0.91	1.00	0.93
noExpAssay		0.78	0.86	0.95	0.98	0.93	0.93
TP53_PROF		0.83	0.87	0.96	0.87	1.00	0.93
DeMask	DMS-based	0.78	0.86	0.95	0.95	0.98	0.93
Envision		0.58	0.75	0.92	0.89	0.96	0.88
VARITY		0.78	0.86	0.95	0.97	0.98	0.93
SIFT	Conservation-based	0.58	0.84	0.85	0.92	0.75	0.98
PolyPhen2		0.47	0.78	0.77	0.96	0.64	0.97
SNAP2		0.59	0.84	0.88	0.95	0.81	0.96
PROVEAN		0.59	0.81	0.90	0.94	0.89	0.92
AlphaMissense	Deep learning–based	0.82	0.89	0.96	0.99	0.98	0.94
ESM-1b		0.52	0.75	0.90	0.87	0.90	0.89
CPT-1		0.63	0.76	0.93	0.95	0.98	0.88

These performance assessments were first benchmarked with the predictions using only experimental data and the imbalance distributions of phenotypes. However, using the experimental data only shows robust performance on the blind test but not on clinical validation, with MCC down to 0.69 (Table S2 available online at http://bib.oxfordjournals.org/), while we got the worse performance (MCC down to 0.44) if we only marked all mutations in DBD as non-functional/pathogenic. (Table S2 available online at http://bib.oxfordjournals.org/). Both these results show the necessity of the other features in both ExpAssay and noExpAssay models.

There are six important hotspot mutation sites (R175, G245, R248, R249, R273 and R282 [43, 90]) in DBD occupying the majority (74%) of cancer cases. Both our ExpAssay and noExpAssay models show correct predictions on all mutations of these residues, suggesting the robustness of our models on hotspot sites. However, the models still got some of the mutations in DBD misclassified (Table S3 available online at http://bib.oxfordjournals.org/) because of the noise in the dataset. Our ExpAssay models failed to predict some of the mutations related to the residue with a structure-breaking side chain (G and P). Wrong classifications also occur more frequently when the mutations have similar residue types, such as D and E, S and T and V/L and F. A small case study (Figure S7 available online at http://bib.oxfordjournals.org/) was performed in the Supporting Information available online at http://bib.oxfordjournals.org/. As for the mutations in the OD, our model failed to predict non-functional mutations. Most of the detrimental mutations in the OD region (Q331, G334, R337 and R342) were misclassified. Only R337P in the clinical validation set was correctly predicted, implying improvement is needed in the future to have features distinguishing pathogenic mutations in the OD.

### Performance comparison with other predictors of mutation effects

Compared with the p53-specific method, TP53_PROF, our ExpAssay presents consistent performance (blind test MCC = 0.88, clinical validation MCC = 0.83, Tables 1 and 2). Meanwhile, we also tried to use the same data splitting of TP53_PROF for model development, and the ExpAssay still achieves stable performance (MCC = 0.87, F1-score = 0.97, Table S4 available online at http://bib.oxfordjournals.org/). However, the ExpAssay model not only has less dependency on the costly experimental assay but also utilizes only nine features for prediction, compared with a more complicated TP53_PROF model containing 42 features. This presents a less concern on ‘over-fitting’ of our model.

In terms of the DMS-based methods and the conventional variant effect predictors, both our ExpAssay and noExpAssay models outperform those tools on both the blind test (Table 1) and on the clinical validation (Table 2), suggesting the insufficient power to predict pathogenic mutations in p53 using the sequence-based generalized features. As for the deep learning-based methods, both our models achieved a better predictive performance on the blind test. On the clinical validation, AlphaMissense achieved a consistent performance with our ExpAssay model, while this method outperformed our noExpAssay model (ExpAssay MCC = 0.83, noExpAssay MCC = 0.78, AlphaMissense MCC = 0.82, Figure S8 available online at http://bib.oxfordjournals.org/). This may suggest the importance of the use of structural features for the characterization of the effects of variants.

### Feature interpretations to improve understanding potential pathogenic risk factors

Since our model could accurately identify deleterious mutations, feature interpretation techniques were deployed to assess the different contributions of each chosen feature and further investigate potential molecular drivers of p53 leading to cancer.

#### Features of ExpAssay model

The highest contributing feature within this model is the fitness score obtained from the experimental assay [79], which was established to identify the dominant negative alleles (Figure 4G). In the case of tetrameric p53, the dominant negative effect may suggest a mutation, even if present in one monomer, may disrupt overall tetramer function, similar to the findings we have reported on PPI binding affinity.

To better understand the experimental fitness score, we computed the Pearson’s correlation coefficient (*R*) between our computational biophysical measurements and the fitness scores from experiment (Table S5 available online at http://bib.oxfordjournals.org/). Notably, the fitness score has a high correlation with the MTRX, a consensus score that distinguishes pathogenic variants from benign ones [62] (*R* = 0.72), suggesting it is also a good measure for pathogenicity. Meanwhile, it also correlated to other biophysical measurements, including the effect of p53 mutations on protein stability (*R* = −0.61 to −0.52), protein–protein interaction affinity (*R* = −0.44 to −0.40) and features describing mutation structural environment, such as the solvent exposure (*R* = −0.68 to −0.61), which suggests that this score also represents effects on function.

Despite the evident contributions of the experimental assay, the other features are also indispensable. The *PP:6.00* generated by graph-based signatures suggests the association of polar atomic environment with non-functional mutations, while the biochemical scores from AAindex [91–94] suggests the importance of protein stability, the change of secondary structure and the conservation of p53. The ionic residue interaction could also be instrumental in characterizing pathogenic mutations.

#### Features of noExpAssay model

Features leveraged by the noExpAssay model show consistent findings of the ExpAssay model and the qualitative comparisons. The top four contributing features describe the effect of mutations comprehensively, and include the local polar atomic environment, the prediction of functional changes (predicted by SNAP2), tolerance to missense mutation estimated by MTR, as well as the mutation impact on protein stability computed by DUET. Without the experimental functionality evaluation, the noExpAssay model used an *in silico* functional prediction as an alternative, showing the strong reliance between function and disease phenotypes. Feature *Pos* captures the pharmacophore change on the positive atom caused by mutations. Additional biochemical scores from AAindex suggest the importance of residue potential [95] and structural similarity of amino acids [96]. Inter-chain hydrophobic and polar residue environments at mutation sites could be useful for the model to classify pathogenic mutations.

### Mutational landscape via computational saturation mutagenesis

**Figure 5:**
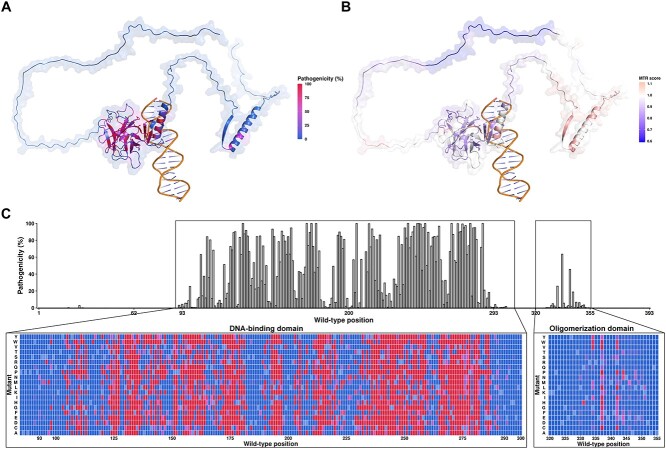
Computational saturation mutagenesis in p53 predicted by our final model (ExpAssay). The pathogenicity of p53, which was coloured based on the confidence of prediction, was mapped to the protein structure (**A**) and the sequence (**C**) of p53, with detailed predictions on the DBD and the OD, while synonymous mutations were coloured as cyan, which was assumed to be non-deleterious. Missense tolerance ratio (MTR) scores were also mapped to the structure (**B**) to display conserved regions of p53.

Finally, we present a comprehensive mutational landscape by employing our best machine learning model (ExpAssay) to all possible missense mutations in p53 (Figure 5). There are approximately 26% predicted-pathogenic mutations in human p53 (Figure S7 available online at http://bib.oxfordjournals.org/), localizing in the DBD and OD. VUS data in the clinical dataset were also annotated using our final model (Figure S9 available online at http://bib.oxfordjournals.org/). All the predictions, along with their confidence estimation, are available in the Supplementary Files available online at http://bib.oxfordjournals.org/.

The p53 mutation landscape can be analysed based on different domains. In our functionality dataset, non-functional mutations were only found in the DBD and the OD. However, our saturation mutagenesis map identifies two mutations (P27Q and P27V) in the TAD with a relatively higher probability (~0.18) to be non-functional. In the DBD, it is predicted that the benign and pathogenic mutations clustered in some of the positions. Some mutation sites, such as T102, H115, A119, S183 and R209, are less likely to harbor mutations having deleterious effects, while the opposite is observed for other positions, such as C132, C176, C238, P278, E286 and R282. In terms of the mutations in OD, several mutations at G334, R337 and R342 should gain attention about the potential pathogenicity. Finally, no pathogenic mutation was found in the CTD.

The mutation landscape of different residue substitutions emphasizes the mutants imparting a high risk of cancer development (Figure S10 available online at http://bib.oxfordjournals.org/). In the DBD region, mutations from Cys, Ile and Phe are more likely to lead to cancer, while mutations from Trp tend to be benign. In terms of hot-spot mutantions, the negatively charged residues (Glu and Asp), along with Pro tend to show a higher probability of functional p53 disruption (Figure S10 available online at http://bib.oxfordjournals.org/).

## DISCUSSION

Tumour suppressor p53, as a leading driver of various cancers, has been extensively studied in the last five decades to identify the sequence–structure–disease relationship. Missense mutations are one of the major alterations of p53 sequence and structure, and it is highly challenging to experimentally elucidate the effects caused by all possible mutations.

Through integrating structural information and leveraging different computational tools, we have sought to characterize the effect of missense mutations in p53. In this work we propose several cancerous risk factors identified from both qualitative comparisons and machine learning analysis, including mutations with dominant negative effects, the changes of protein stability and PPI binding affinity and the polar environment at the mutation site. While the effects of mutations on protein–DNA binding affinity did not show any significance, mutations directly altering DNA binding were the minority [97], masking this effect. Reassuringly, however, the final predictive model was able to correctly capture the phenotypic outcomes of the high-frequency TP53 disease mutations previously shown to alter DNA binding. These molecular drivers have also been studied experimentally in previous works [89, 98, 99], showing the robustness of our analysis. Further to that, the feature interpretation techniques such as SHAP largely improve the understanding of these molecular drivers. For example, it is presented that the destabilizing effect caused by mutations is strongly associated with deleterious consequences on p53 function, suggesting that p53 function can be restored by stabilizing its mutant structure. Recent work has also shown that using a small molecule drug via virtual screening could help stabilize the mutated p53 structures and recover its functions [43]. We therefore believe that our proposed molecular drivers could guide new strategies to design personalized treatments.

The main feature contributing to our final model is one of the functional fitness scores generated by an experimental assay. However, it should be emphasized that the precise experimental assay can be used for characterization of functional changes but not the clinical pathogenicity (Table S1 available online at http://bib.oxfordjournals.org/). With a comparable performance, our noExpAssay model using computational measurements has less dependency on the costly experiments. The power of these *in silico* features has been well validated on other proteins [37–39]. For the purpose of clinical use, this would justify the inclusion of experimental assays, if available. On the other hand, the computational features may have a wider applicability and better interpretation of the model.

Both our method and the state-of-the-art approaches could accurately classify the cancer-causing mutations on the DBD but showed performance deterioration on mutations in the OD, due to the rare occurrence of mutations in this domain. Further to that, many experimental assays focused on estimating the fitness of mutation only in the DBD [97, 100, 101] rather than the other domains. Though these functionality assessments could largely help improve the predictive performance of the identification of deleterious mutations [36], more specific features could be developed to improve the specificity of the mutations of OD, as the pathogenic variants in OD could have different effects on p53 monomer, dimer and tetramer [8]. We attempted to use the whole p53 structure as the input to try to provide sufficient structural information for feature generation, but the contributions were limited, with only one mutation accurately predicted. Further improvement would be needed for the variants in this domain.

In brief, our model could accurately characterize p53 cancer-causing missense mutations by combining large-scale experimental assay results, protein structural information and various computational tools. These accurate predictions could be invaluable for the research on p53 and cancer.

**Key Points**

- We established two interpretable machine learning models accurately identifying cancerous missense mutations of the tumour suppressor p53 with comparable performance of the state-of-the-art methods.
- Qualitative analysis and model interpretation revealed that information on residual p53 activity, polar atomic environment and changes in p53 stability were instrumental in the decisions.
- Computational saturation mutagenesis of p53 was generated for patient monitoring and the development of cancer treatments.

## Supplementary Material

p53_qishengpan_supps_bbad428

p53_af2_bbad428

clinical_validation_bbad428

## Data Availability

The functionality dataset for model development is available in the paper of TP53_PROF at https://doi.org/10.1093/bib/bbab524. The clinical validation, the p53 tetramer generated by AlphaFold2, and results of the saturation mutagenesis are available in the Supplementary Files available online at http://bib.oxfordjournals.org/. Fitness scores generated by the experimental assays are available in the paper at https://doi.org/10.1038/s41588-018-0204-y. The use of computational biophysical measurements is available by running on the web servers through https://biosig.lab.uq.edu.au/tools. The other computational features could be found according to the reference publications. The source code for statistical tests and machine learning analysis was available at: https://github.com/Sunny8192/p53_mutation_analysis/tree/main.

## Author Biographies

**Qisheng Pan** is a PhD candidate at the School of Chemistry and Molecular Bioscience of the University of Queensland. His research interestsinclude characterising and predicting the effects of mutation on protein function and structure.

**Stephanie Portelli** is a postdoctoral research fellow at the School of Chemistry and Molecular Bioscience of the University of Queensland. Her research focuses on the characterisation of clinical reported variants by leveraging computational mutation analysis and machine learning.

**Thanh Binh Nguyen** is a postdoctoral research fellow at the School of Chemistry and Molecular Bioscience of the University of Queensland. Her interest is in computational biology and understanding protein structures, function and their interactions.

**David B. Ascher** is the Director of the Biotechnology Program at The University of Queensland, and head of Computational Biology and Clinical Informatics at the Baker Institute. He is interested in developing and applying computational tools to assist leveraging clinical and omics data for drug discovery and personalised medicine.

## References

[1] Hollstein M, Sidransky D, Vogelstein B, Harris CC. p53 mutations in human cancers. Science 1991;253:49–53. 1905840 10.1126/science.1905840

[2] Hamroun D, Kato S, Ishioka C, et al. The UMD TP53 database and website: update and revisions. Hum Mutat 2006;27:14–20. 16278824 10.1002/humu.20269

[3] Olivier M, Eeles R, Hollstein M, et al. The IARC TP53 database: new online mutation analysis and recommendations to users. Hum Mutat 2002;19:607–14. 12007217 10.1002/humu.10081

[4] Petitjean A, Mathe E, Kato S, et al. Impact of mutant p53 functional properties on TP53 mutation patterns and tumor phenotype: lessons from recent developments in the IARC TP53 database. Hum Mutat 2007;28:622–9. 17311302 10.1002/humu.20495

[5] Baugh EH, Ke H, Levine AJ, et al. Why are there hotspot mutations in the TP53 gene in human cancers? Cell Death Differ 2018;25:154–60. 29099487 10.1038/cdd.2017.180 PMC5729536

[6] Guha T, Malkin D. Inherited TP53 mutations and the Li-Fraumeni syndrome. Cold Spring Harb Perspect Med 2017;7(4):a026187. 28270529 10.1101/cshperspect.a026187 PMC5378014

[7] Laptenko O, Shiff I, Freed-Pastor W, et al. The p53 C terminus controls site-specific DNA binding and promotes structural changes within the central DNA binding domain. Mol Cell 2015;57:1034–46. 25794615 10.1016/j.molcel.2015.02.015 PMC6221458

[8] Fischer NW, Prodeus A, Malkin D, Gariepy J. p53 oligomerization status modulates cell fate decisions between growth, arrest and apoptosis. Cell Cycle 2016;15:3210–9. 27754743 10.1080/15384101.2016.1241917 PMC5176156

[9] Golovenko D, Brauning B, Vyas P, et al. New insights into the role of DNA shape on its recognition by p53 proteins. Structure 2018;26:1237, e1236–50. 30057026 10.1016/j.str.2018.06.006

[10] Zhao J, Liu X, Blayney A, et al. Intrinsically disordered N-terminal domain (NTD) of p53 interacts with mitochondrial PTP regulator Cyclophilin D. J Mol Biol 2022;434:167552. 10.1016/j.jmb.2022.167552. 35341741

[11] Wang P, Reed M, Wang Y, et al. p53 domains: structure, oligomerization, and transformation. Mol Cell Biol 1994;14:5182–91. 8035799 10.1128/mcb.14.8.5182-5191.1994 PMC359037

[12] Zhao J, Blayney A, Liu X, et al. EGCG binds intrinsically disordered N-terminal domain of p53 and disrupts p53-MDM2 interaction. Nat Commun 2021;12:986. 33579943 10.1038/s41467-021-21258-5 PMC7881117

[13] Hirao A, Kong YY, Matsuoka S, et al. DNA damage-induced activation of p53 by the checkpoint kinase Chk2. Science 2000;287:1824–7. 10710310 10.1126/science.287.5459.1824

[14] Di Leonardo A, Linke SP, Clarkin K, Wahl GM. DNA damage triggers a prolonged p53-dependent G1 arrest and long-term induction of Cip1 in normal human fibroblasts. Genes Dev 1994;8:2540–51. 7958916 10.1101/gad.8.21.2540

[15] Kastan MB, Zhan Q, El-Deiry WS, et al. A mammalian cell cycle checkpoint pathway utilizing p53 and GADD45 is defective in ataxia-telangiectasia. Cell 1992;71:587–97. 1423616 10.1016/0092-8674(92)90593-2

[16] Wachter F, Grunert M, Blaj C, et al. Impact of the p53 status of tumor cells on extrinsic and intrinsic apoptosis signaling. Cell Commun Signal 2013;11:27. 23594441 10.1186/1478-811X-11-27 PMC3641951

[17] Wang P, Yu J, Zhang L. The nuclear function of p53 is required for PUMA-mediated apoptosis induced by DNA damage. Proc Natl Acad Sci U S A 2007;104:4054–9. 17360476 10.1073/pnas.0700020104 PMC1820707

[18] Feng Z, Liu L, Zhang C, et al. Chronic restraint stress attenuates p53 function and promotes tumorigenesis. Proc Natl Acad Sci U S A 2012;109:7013–8. 22509031 10.1073/pnas.1203930109 PMC3345015

[19] Shi T, van Soest DMK, Polderman PE, et al. DNA damage and oxidant stress activate p53 through differential upstream signaling pathways. Free Radic Biol Med 2021;172:298–311. 34144191 10.1016/j.freeradbiomed.2021.06.013

[20] Kussie PH, Gorina S, Marechal V, et al. Structure of the MDM2 oncoprotein bound to the p53 tumor suppressor transactivation domain. Science 1996;274:948–53. 8875929 10.1126/science.274.5289.948

[21] Schumacher B, Hofmann K, Boulton S, Gartner A. The C. elegans homolog of the p53 tumor suppressor is required for DNA damage-induced apoptosis. Curr Biol 2001;11:1722–7. 11696333 10.1016/s0960-9822(01)00534-6

[22] Derry WB, Putzke AP, Rothman JH. Caenorhabditis elegans p53: role in apoptosis, meiosis, and stress resistance. Science 2001;294:591–5. 11557844 10.1126/science.1065486

[23] Han J, Goldstein LA, Hou W, et al. Regulation of mitochondrial apoptotic events by p53-mediated disruption of complexes between antiapoptotic Bcl-2 members and Bim. J Biol Chem 2010;285:22473–83. 20404322 10.1074/jbc.M109.081042 PMC2903343

[24] Shibue T, Takeda K, Oda E, et al. Integral role of Noxa in p53-mediated apoptotic response. Genes Dev 2003;17:2233–8. 12952892 10.1101/gad.1103603 PMC196460

[25] Trinidad AG, Muller PA, Cuellar J, et al. Interaction of p53 with the CCT complex promotes protein folding and wild-type p53 activity. Mol Cell 2013;50:805–17. 23747015 10.1016/j.molcel.2013.05.002 PMC3699784

[26] Bullock AN, Henckel J, DeDecker BS, et al. Thermodynamic stability of wild-type and mutant p53 core domain. Proc Natl Acad Sci U S A 1997;94:14338–42. 9405613 10.1073/pnas.94.26.14338 PMC24967

[27] Pavletich NP, Chambers KA, Pabo CO. The DNA-binding domain of p53 contains the four conserved regions and the major mutation hot spots. Genes Dev 1993;7:2556–64. 8276238 10.1101/gad.7.12b.2556

[28] Kandoth C, McLellan MD, Vandin F, et al. Mutational landscape and significance across 12 major cancer types. Nature 2013;502:333–9. 24132290 10.1038/nature12634 PMC3927368

[29] Hwang HJ, Nam SK, Park H, et al. Prediction of TP53 mutations by p53 immunohistochemistry and their prognostic significance in gastric cancer. J Pathol Transl Med 2020;54:378–86. 32601264 10.4132/jptm.2020.06.01 PMC7483024

[30] Xu F, Lin H, He P, et al. A TP53-associated gene signature for prediction of prognosis and therapeutic responses in lung squamous cell carcinoma. Onco Targets Ther 2020;9:1731943. 10.1080/2162402X.2020.1731943 PMC7051188 32158625

[31] Shi C, Liu S, Tian X, et al. A TP53 mutation model for the prediction of prognosis and therapeutic responses in head and neck squamous cell carcinoma. BMC Cancer 2021;21:1035. 34530752 10.1186/s12885-021-08765-w PMC8447564

[32] Giacomelli AO, Yang X, Lintner RE, et al. Mutational processes shape the landscape of TP53 mutations in human cancer. Nat Genet 2018;50:1381–7. 30224644 10.1038/s41588-018-0204-y PMC6168352

[33] Choi Y, Chan AP. PROVEAN web server: a tool to predict the functional effect of amino acid substitutions and indels. Bioinformatics 2015;31:2745–7. 25851949 10.1093/bioinformatics/btv195 PMC4528627

[34] Adzhubei I, Jordan DM, Sunyaev SR. Predicting functional effect of human missense mutations using PolyPhen-2. Curr Protoc Hum Genet Chapter 7 2013;76:Unit7.20. 10.1002/0471142905.hg0720s76 PMC4480630 23315928

[35] Ng PC, Henikoff S. Predicting deleterious amino acid substitutions. Genome Res 2001;11:863–74. 11337480 10.1101/gr.176601 PMC311071

[36] Ben-Cohen G, Doffe F, Devir M, et al. TP53_PROF: a machine learning model to predict impact of missense mutations in TP53. Brief Bioinform 2022;23:bbab524. 10.1093/bib/bbab524. PMC8921628 35043155

[37] Portelli S, Albanaz A, Pires DEV, Ascher DB. Identifying the molecular drivers of ALS-implicated missense mutations. J Med Genet 2023;60:484–90. 36180205 10.1136/jmg-2022-108798

[38] Portelli S, Barr L, de Sa AGC, et al. Distinguishing between PTEN clinical phenotypes through mutation analysis. Comput Struct Biotechnol J 2021;19:3097–109. 34141133 10.1016/j.csbj.2021.05.028 PMC8180946

[39] Aljarf R, Shen M, Pires DEV, Ascher DB. Understanding and predicting the functional consequences of missense mutations in BRCA1 and BRCA2. Sci Rep 2022;12:10458. 35729312 10.1038/s41598-022-13508-3 PMC9213547

[40] Portelli S, Phelan JE, Ascher DB, et al. Understanding molecular consequences of putative drug resistant mutations in *Mycobacterium tuberculosis*. Sci Rep 2018;8:15356. 30337649 10.1038/s41598-018-33370-6 PMC6193939

[41] Zhou Y, Portelli S, Pat M, et al. Structure-guided machine learning prediction of drug resistance mutations in Abelson 1 kinase. Comput Struct Biotechnol J 2021;19:5381–91. 34667533 10.1016/j.csbj.2021.09.016 PMC8495037

[42] Portelli S, Myung Y, Furnham N, et al. Prediction of rifampicin resistance beyond the RRDR using structure-based machine learning approaches. Sci Rep 2020;10:18120. 33093532 10.1038/s41598-020-74648-y PMC7581776

[43] Durairaj G, Demir O, Lim B, et al. Discovery of compounds that reactivate p53 mutants in vitro and in vivo. Cell Chem Biol 2022;29:1381, e1313–1395.e13. 35948006 10.1016/j.chembiol.2022.07.003 PMC9481737

[44] Beroud C, Soussi T. The UMD-p53 database: new mutations and analysis tools. Hum Mutat 2003;21:176–81. 12619103 10.1002/humu.10187

[45] Cancer Genome Atlas Research, N, Weinstein JN, Collisson EA, et al. The cancer genome atlas Pan-cancer analysis project. Nat Genet 2013;45:1113–20. 24071849 10.1038/ng.2764 PMC3919969

[46] Cheng DT, Mitchell TN, Zehir A, et al. Memorial Sloan Kettering-integrated mutation profiling of actionable cancer targets (MSK-IMPACT): a hybridization capture-based next-generation sequencing clinical assay for solid tumor molecular oncology. J Mol Diagn 2015;17:251–64. 25801821 10.1016/j.jmoldx.2014.12.006 PMC5808190

[47] International Cancer Genome, C, Hudson TJ, Anderson W, et al. International network of cancer genome projects. Nature 2010;464:993–8. 20393554 10.1038/nature08987 PMC2902243

[48] Landrum MJ, Lee JM, Benson M, et al. ClinVar: improving access to variant interpretations and supporting evidence. Nucleic Acids Res 2018;46:D1062–7. 29165669 10.1093/nar/gkx1153 PMC5753237

[49] Richards S, Aziz N, Bale S, et al. Standards and guidelines for the interpretation of sequence variants: a joint consensus recommendation of the American College of Medical Genetics and Genomics and the Association for Molecular Pathology. Genet Med 2015;17:405–24. 25741868 10.1038/gim.2015.30 PMC4544753

[50] Fortuno C, Lee K, Olivier M, et al. Specifications of the ACMG/AMP variant interpretation guidelines for germline TP53 variants. Hum Mutat 2021;42:223–36. 33300245 10.1002/humu.24152 PMC8374922

[51] Jumper J, Evans R, Pritzel A, et al. Highly accurate protein structure prediction with AlphaFold. Nature 2021;596:583–9. 34265844 10.1038/s41586-021-03819-2 PMC8371605

[52] Pan Q, Nguyen TB, Ascher DB, Pires DEV. Systematic evaluation of computational tools to predict the effects of mutations on protein stability in the absence of experimental structures. Brief Bioinform 2022;23:bbac025. 10.1093/bib/bbac025. PMC9155634 35189634

[53] Richard E, Michael ON, Alexander P, et al. Protein complex prediction with AlphaFold-Multimer. bioRxiv 2021.2010.2004.463034 2022. 10.1101/2021.10.04.463034.

[54] Mariani V, Biasini M, Barbato A, Schwede T. lDDT: a local superposition-free score for comparing protein structures and models using distance difference tests. Bioinformatics 2013;29:2722–8. 23986568 10.1093/bioinformatics/btt473 PMC3799472

[55] Jeffrey PD, Gorina S, Pavletich NP. Crystal structure of the tetramerization domain of the p53 tumor suppressor at 1.7 angstroms. Science 1995;267:1498–502. 7878469 10.1126/science.7878469

[56] Waterman JL, Shenk JL, Halazonetis TD. The dihedral symmetry of the p53 tetramerization domain mandates a conformational switch upon DNA binding. EMBO J 1995;14:512–9. 7859740 10.1002/j.1460-2075.1995.tb07027.x PMC398109

[57] Kawashima S, Ogata H, Kanehisa M. AAindex: amino acid index database. Nucleic Acids Res 1999;27:368–9. 9847231 10.1093/nar/27.1.368 PMC148186

[58] Altschul SF, Madden TL, Schaffer AA, et al. Gapped BLAST and PSI-BLAST: a new generation of protein database search programs. Nucleic Acids Res 1997;25:3389–402. 9254694 10.1093/nar/25.17.3389 PMC146917

[59] Camacho C, Coulouris G, Avagyan V, et al. BLAST+: architecture and applications. BMC Bioinformatics 2009;10:421. 20003500 10.1186/1471-2105-10-421 PMC2803857

[60] Sayers EW, Bolton EE, Brister JR, et al. Database resources of the national center for biotechnology information. Nucleic Acids Res 2022;50:D20–6. 34850941 10.1093/nar/gkab1112 PMC8728269

[61] Silk M, Petrovski S, Ascher DB. MTR-viewer: identifying regions within genes under purifying selection. Nucleic Acids Res 2019;47:W121–6. 31170280 10.1093/nar/gkz457 PMC6602522

[62] Silk M, Pires DEV, Rodrigues CHM, et al. MTR3D: identifying regions within protein tertiary structures under purifying selection. Nucleic Acids Res 2021;49:W438–45. 34050760 10.1093/nar/gkab428 PMC8265191

[63] Cock PJ, Antao T, Chang JT, et al. Biopython: freely available python tools for computational molecular biology and bioinformatics. Bioinformatics 2009;25:1422–3. 19304878 10.1093/bioinformatics/btp163 PMC2682512

[64] Joosten RP, te Beek TA, Krieger E, et al. A series of PDB related databases for everyday needs. Nucleic Acids Res 2011;39:D411–9. 21071423 10.1093/nar/gkq1105 PMC3013697

[65] Kabsch W, Sander C. Dictionary of protein secondary structure: pattern recognition of hydrogen-bonded and geometrical features. Biopolymers 1983;22:2577–637. 6667333 10.1002/bip.360221211

[66] Sali A, Blundell TL. Comparative protein modelling by satisfaction of spatial restraints. J Mol Biol 1993;234:779–815. 8254673 10.1006/jmbi.1993.1626

[67] Marti-Renom MA, Stuart AC, Fiser A, et al. Comparative protein structure modeling of genes and genomes. Annu Rev Biophys Biomol Struct 2000;29:291–325. 10940251 10.1146/annurev.biophys.29.1.291

[68] Webb B, Sali A. Comparative protein structure modeling using MODELLER. Curr Protoc Bioinformatics 2016;54:5.6.1–37. 10.1002/cpbi.3 PMC5031415 27322406

[69] Jubb HC, Higueruelo AP, Ochoa-Montano B, et al. Arpeggio: a web server for calculating and visualising interatomic interactions in protein structures. J Mol Biol 2017;429:365–71. 27964945 10.1016/j.jmb.2016.12.004 PMC5282402

[70] Pires DE, Ascher DB, Blundell TL. mCSM: predicting the effects of mutations in proteins using graph-based signatures. Bioinformatics 2014;30:335–42. 24281696 10.1093/bioinformatics/btt691 PMC3904523

[71] Pires DE, Ascher DB, Blundell TL. DUET: a server for predicting effects of mutations on protein stability using an integrated computational approach. Nucleic Acids Res 2014;42:W314–9. 24829462 10.1093/nar/gku411 PMC4086143

[72] Pandurangan AP, Ochoa-Montano B, Ascher DB, Blundell TL. SDM: a server for predicting effects of mutations on protein stability. Nucleic Acids Res 2017;45:W229–35. 28525590 10.1093/nar/gkx439 PMC5793720

[73] Frappier V, Chartier M, Najmanovich RJ. ENCoM server: exploring protein conformational space and the effect of mutations on protein function and stability. Nucleic Acids Res 2015;43:W395–400. 25883149 10.1093/nar/gkv343 PMC4489264

[74] Rodrigues CH, Pires DE, Ascher DB. DynaMut: predicting the impact of mutations on protein conformation, flexibility and stability. Nucleic Acids Res 2018;46:W350–5. 29718330 10.1093/nar/gky300 PMC6031064

[75] Rodrigues CHM, Pires DEV, Ascher DB. DynaMut2: assessing changes in stability and flexibility upon single and multiple point missense mutations. Protein Sci 2021;30:60–9. 32881105 10.1002/pro.3942 PMC7737773

[76] Li G, Panday SK, Alexov E. SAAFEC-SEQ: a sequence-based method for predicting the effect of single point mutations on protein thermodynamic stability. Int J Mol Sci 2021;22:606. 10.3390/ijms22020606. PMC7827184 33435356

[77] Rodrigues CHM, Myung Y, Pires DEV, Ascher DB. mCSM-PPI2: predicting the effects of mutations on protein-protein interactions. Nucleic Acids Res 2019;47:W338–44. 31114883 10.1093/nar/gkz383 PMC6602427

[78] Pires DEV, Ascher DB. mCSM-NA: predicting the effects of mutations on protein-nucleic acids interactions. Nucleic Acids Res 2017;45:W241–6. 28383703 10.1093/nar/gkx236 PMC5570212

[79] Klinakis A, Rampias T. TP53 mutational landscape of metastatic head and neck cancer reveals patterns of mutation selection. EBioMedicine 2020;58:102905. 32739866 10.1016/j.ebiom.2020.102905 PMC7393519

[80] Hecht M, Bromberg Y, Rost B. Better prediction of functional effects for sequence variants. BMC Genomics 2015;16(Suppl 8):S1. 10.1186/1471-2164-16-S8-S1 PMC4480835 26110438

[81] Myung Y, Pires DEV, Ascher DB. mmCSM-AB: guiding rational antibody engineering through multiple point mutations. Nucleic Acids Res 2020;48:W125–31. 32432715 10.1093/nar/gkaa389 PMC7319589

[82] Lundberg SM, Erion G, Chen H, et al. From local explanations to global understanding with explainable AI for trees. Nat Mach Intell 2020;2:56–67. 32607472 10.1038/s42256-019-0138-9 PMC7326367

[83] Munro D, Singh M. DeMaSk: a deep mutational scanning substitution matrix and its use for variant impact prediction. Bioinformatics 2021;36:5322–9. 33325500 10.1093/bioinformatics/btaa1030 PMC8016454

[84] Gray VE, Hause RJ, Luebeck J, et al. Quantitative missense variant effect prediction using large-scale mutagenesis data. Cell Syst 2018;6:116, e113–24. 29226803 10.1016/j.cels.2017.11.003 PMC5799033

[85] Wu Y, Liu H, Li R, et al. Improved pathogenicity prediction for rare human missense variants. Am J Hum Genet 2021;108:2389. 34861178 10.1016/j.ajhg.2021.11.010 PMC8715197

[86] Cheng J, Novati G, Pan J, et al. Accurate proteome-wide missense variant effect prediction with AlphaMissense. Science 2023;381:eadg7492. 37733863 10.1126/science.adg7492

[87] Brandes N, Goldman G, Wang CH, et al. Genome-wide prediction of disease variant effects with a deep protein language model. Nat Genet 2023;55:1512–22. 37563329 10.1038/s41588-023-01465-0 PMC10484790

[88] Jagota M, Ye C, Albors C, et al. Cross-protein transfer learning substantially improves disease variant prediction. Genome Biol 2023;24:182. 37550700 10.1186/s13059-023-03024-6 PMC10408151

[89] Blanden AR, Yu X, Blayney AJ, et al. Zinc shapes the folding landscape of p53 and establishes a pathway for reactivating structurally diverse cancer mutants. Elife 2020;9:e61487. 10.7554/eLife.61487. PMC7728444 33263541

[90] Cho Y, Gorina S, Jeffrey PD, Pavletich NP. Crystal structure of a p53 tumor suppressor-DNA complex: understanding tumorigenic mutations. Science 1994;265:346–55. 8023157 10.1126/science.8023157

[91] Boniecki M, Rotkiewicz P, Skolnick J, Kolinski A. Protein fragment reconstruction using various modeling techniques. J Comput Aided Mol Des 2003;17:725–38. 15072433 10.1023/b:jcam.0000017486.83645.a0

[92] Blake JD, Cohen FE. Pairwise sequence alignment below the twilight zone. J Mol Biol 2001;307:721–35. 11254392 10.1006/jmbi.2001.4495

[93] Mehta PK, Heringa J, Argos P. A simple and fast approach to prediction of protein secondary structure from multiply aligned sequences with accuracy above 70%. Protein Sci 1995;4:2517–25. 8580842 10.1002/pro.5560041208 PMC2143048

[94] Gianese G, Argos P, Pascarella S. Structural adaptation of enzymes to low temperatures. Protein Eng 2001;14:141–8. 11342709 10.1093/protein/14.3.141

[95] Micheletti C, Seno F, Banavar JR, Maritan A. Learning effective amino acid interactions through iterative stochastic techniques. Proteins 2001;42:422–31. 11151013 10.1002/1097-0134(20010215)42:3<422::aid-prot120>3.0.co;2-2

[96] Feng DF, Johnson MS, Doolittle RF. Aligning amino acid sequences: comparison of commonly used methods. J Mol Evol 1984;21:112–25. 6100188 10.1007/BF02100085

[97] Kato S, Han SY, Liu W, et al. Understanding the function-structure and function-mutation relationships of p53 tumor suppressor protein by high-resolution missense mutation analysis. Proc Natl Acad Sci U S A 2003;100:8424–9. 12826609 10.1073/pnas.1431692100 PMC166245

[98] Gencel-Augusto J, Lozano G. p53 tetramerization: at the center of the dominant-negative effect of mutant p53. Genes Dev 2020;34:1128–46. 32873579 10.1101/gad.340976.120 PMC7462067

[99] Khadiullina R, Mirgayazova R, Davletshin D, et al. Assessment of thermal stability of mutant p53 proteins via differential scanning Fluorimetry. Life (Basel) 2022;13(1):31. 10.3390/life13010031. 36675980 PMC9862671

[100] Carbonnier V, Leroy B, Rosenberg S, Soussi T. Comprehensive assessment of TP53 loss of function using multiple combinatorial mutagenesis libraries. Sci Rep 2020;10:20368. 33230179 10.1038/s41598-020-74892-2 PMC7683535

[101] Kotler E, Shani O, Goldfeld G, et al. A systematic p53 mutation library links differential functional impact to cancer mutation pattern and evolutionary conservation. Mol Cell 2018;71:178, e178–90. 29979965 10.1016/j.molcel.2018.06.012

