# Supplementary material for: Characterization on the oncogenic effect of the missense mutations of p53 via machine learning
Source: Brief Bioinform. 2023 Nov 28;25(1):bbad428. doi: 10.1093/bib/bbad428 (PMC10685404; doi:10.1093/bib/bbad428)
Supplement: p53_qishengpan_supps_bbad428 [file p53_qishengpan_supps_bbad428.docx]

**Supporting Information**

**Characterisation on the oncogenic effect of the missense mutations of p53 via machine learning**

Qisheng Pan^1,2^, Stephanie Portelli^1,2^, Thanh Binh Nguyen^1,2^, David B. Ascher^1,2,*^

^1^School of Chemistry and Molecular Bioscience, University of Queensland, Brisbane Queensland 4072, Australia

^2^Computational Biology and Clinical Informatics, Baker Heart and Diabetes Institute, Melbourne Victoria 3004, Australia

*To whom correspondence should be addressed to D.B.A. Tel: +61 7 336 53991; Email: [d.ascher@uq.edu.au](mailto:d.ascher@uq.edu.au).

**TABLES**

**Table S1.** Confusion matrices of blind test of ExpAssay and noExpAssay models

| **ExpAssay model** | Actual functional | Actual non-functional |
| --- | --- | --- |
| Predicted functional | 194 | 8 |
| Predicted non-functional | 2 | 47 |
|  |  |  |
| **noExpAssay model** | Actual functional | Actual non-functional |
| Predicted functional | 193 | 13 |
| Predicted non-functional | 3 | 42 |

**Table S2.** Baseline performance using the experimental assay or domain information respectively as features.

| Test dataset | Feature | MCC | BACC | F1-score | Recall | Precision |
| --- | --- | --- | --- | --- | --- | --- |
| Blind test | Experimental assay - cutoff 0.81^a^ | 0.87 | 0.92 | 0.97 | 0.98 | 0.96 |
| Clinical validation | Experimental assay - cutoff 0.81^a^ | 0.69 | 0.77 | 0.94 | 1.00 | 0.88 |
| Blind test | DNA-binding domain information^b^ | 0.61 | 0.87 | 0.85 | 1.00 | 0.96 |
| Clinical validation | DNA-binding domain information^b^ | 0.44 | 0.76 | 0.68 | 0.52 | 1.00 |

^a^Cutoff was generated by optimising the MCC on the training set.

^b^Mark all mutations in this domain as non-functional/pathogenic, and mark the rest of the mutations as benign.

**Table S3.** Wrong predictions of the ExpAssay models in the 10-CV, blind test, and the clinical validation

| Test set | Wrong mutation | Actual label | Predicted label |
| --- | --- | --- | --- |
| 10-CV | P98R | Functional | Non-functional |
| 10-CV | G105C | Non-functional | Functional |
| 10-CV | G105D | Non-functional | Functional |
| 10-CV | G105V | Non-functional | Functional |
| 10-CV | S106R | Non-functional | Functional |
| 10-CV | S121A | Functional | Non-functional |
| 10-CV | C124F | Functional | Non-functional |
| 10-CV | T150S | Functional | Non-functional |
| 10-CV | P151R | Non-functional | Functional |
| 10-CV | P151T | Non-functional | Functional |
| 10-CV | G154R | Functional | Non-functional |
| 10-CV | V157F | Non-functional | Functional |
| 10-CV | A161S | Non-functional | Functional |
| 10-CV | H168Q | Functional | Non-functional |
| 10-CV | E180V | Functional | Non-functional |
| 10-CV | I195V | Functional | Non-functional |
| 10-CV | E224D | Non-functional | Functional |
| 10-CV | E224Q | Functional | Non-functional |
| 10-CV | C229W | Functional | Non-functional |
| 10-CV | M246I | Non-functional | Functional |
| 10-CV | T256R | Functional | Non-functional |
| 10-CV | L265R | Non-functional | Functional |
| 10-CV | R267Q | Non-functional | Functional |
| 10-CV | N268T | Functional | Non-functional |
| 10-CV | F270L | Non-functional | Functional |
| 10-CV | E271K | Non-functional | Functional |
| 10-CV | A276G | Non-functional | Functional |
| 10-CV | G279A | Functional | Non-functional |
| 10-CV | D281E | Non-functional | Functional |
| 10-CV | D281V | Non-functional | Functional |
| 10-CV | E287D | Non-functional | Functional |
| 10-CV | L289F | Non-functional | Functional |
| 10-CV | R290C | Non-functional | Functional |
| 10-CV | Q331H | Non-functional | Functional |
| 10-CV | G334V | Non-functional | Functional |
| 10-CV | R337C | Non-functional | Functional |
| 10-CV | R337L | Non-functional | Functional |
| 10-CV | F338S | Functional | Non-functional |
| 10-CV | R342P | Non-functional | Functional |
| Blind test | T150S | Functional | Non-functional |
| Blind test | P151T | Non-functional | Functional |
| Blind test | V157F | Non-functional | Functional |
| Blind test | E180V | Functional | Non-functional |
| Blind test | M246I | Non-functional | Functional |
| Blind test | D281E | Non-functional | Functional |
| Blind test | D281V | Non-functional | Functional |
| Blind test | E287D | Non-functional | Functional |
| Blind test | G334V | Non-functional | Functional |
| Blind test | R342P | Non-functional | Functional |
| Clinical validation | M160I | Non-functional | Functional |
| Clinical validation | G334W | Non-functional | Functional |
| Clinical validation | R337H | Non-functional | Functional |
| Clinical validation | L344P | Non-functional | Functional |

**Table S4.** Predictive performance of the functional outcome of missense mutations on the blind test described in TP53_PROF.

| Models | MCC | BACC | F1-score | AUC | Recall | Precision |
| --- | --- | --- | --- | --- | --- | --- |
| ExpAssay - TP53_PROF split | 0.87 | 0.93 | 0.97 | 0.99 | 0.98 | 0.97 |
| noExpAssay - TP53_PROF split | 0.74 | 0.87 | 0.94 | 0.95 | 0.94 | 0.95 |
| TP53_PROF (functional features) | 0.90 | 0.95 | 0.92 | 0.97 | 0.93 | 0.91 |
| TP53_PROF (computational features) | 0.74 | 0.90 | 0.80 | 0.96 | 0.89 | 0.73 |
| TP53_PROF (combined) | 0.88 | 0.94 | 0.90 | 0.99 | 0.91 | 0.90 |

**Table S5.** Pearson’s Correlation Coefficient (R) between biophysical measurements and the fitness scores generated by the experimental assay.

| Experimental assay | Feature | R | Description of feature |
| --- | --- | --- | --- |
| A549_p53WT_Nutlin-3_Z-score | MTRX | 0.720 | A consensus score to distinguishes pathogenic from benign variants, which is available on https://biosig.lab.uq.edu.au/mtr3d/ |
| A549_p53WT_Nutlin-3_Z-score | Distance to Zn | -0.680 | Distance from a mutation to the Zn atom |
| A549_p53WT_Nutlin-3_Z-score | RSA | -0.637 | Relative solvent accessibility of a mutation |
| A549_p53WT_Nutlin-3_Z-score | pLDDT | 0.633 | AlphaFold2 confidence score |
| A549_p53WT_Nutlin-3_Z-score | mCSM ΔΔG | -0.614 | Effect of mutation on protein stability computed using mCSM-Stability |
| A549_p53WT_Nutlin-3_Z-score | Residue Depth | 0.612 | A solvent exposure measurement that describes to what extent a residue is buried in protein structure |
| A549_p53WT_Nutlin-3_Z-score | DUET ΔΔG | -0.592 | Effect of mutation on protein stability computed using DUET |
| A549_p53WT_Nutlin-3_Z-score | Distance to dsDNA | -0.558 | Distance from mutation to the dsDNA structure |
| A549_p53WT_Nutlin-3_Z-score | DynaMut2 ΔΔG | -0.519 | Effect of mutation on protein stability computed using DynaMut2 |
| A549_p53WT_Nutlin-3_Z-score | mCSM-PPI1 (monomer B&C) ΔΔG | -0.443 | Effect of mutation on protein-protein interaction (PPI) binding computed using mCSM-PPI1 |
| A549_p53WT_Nutlin-3_Z-score | mCSM-PPI2 (monomer B&C) ΔΔG | -0.428 | Effect of mutation on PPI binding computed using mCSM-PPI2 |
| A549_p53WT_Nutlin-3_Z-score | mCSM-PPI1 (monomer B&D) ΔΔG | -0.410 | Effect of mutation on PPI binding computed using mCSM-PPI1 |
| A549_p53WT_Nutlin-3_Z-score | mCSM-PPI2 (monomer B&D) ΔΔG | -0.400 | Effect of mutation on PPI binding computed using mCSM-PPI2 |
| A549_p53WT_Nutlin-3_Z-score | SDM ΔΔG | -0.375 | Effect of mutation on protein stability computed using SDM |
| A549_p53WT_Nutlin-3_Z-score | SAAFEC-SEQ ΔΔG | -0.355 | Effect of mutation on protein stability computed using SAAFEC-SEQ |
| A549_p53WT_Nutlin-3_Z-score | PPI distance to monomer A | -0.328 | Distance from mutation to the PPI interface |
| A549_p53WT_Nutlin-3_Z-score | MTR3D (8 Å window size) | -0.317 | Missense Tolerance Ratio, available on https://biosig.lab.uq.edu.au/mtr3d/ |
| A549_p53WT_Nutlin-3_Z-score | DynaMut1 ΔΔG | -0.311 | Effect of mutation on protein stability computed using DynaMut1 |
| A549_p53WT_Nutlin-3_Z-score | PPI distance to monomer C | -0.234 | Distance from mutation to the PPI interface |
| A549_p53WT_Nutlin-3_Z-score | PPI distance to monomer D | -0.226 | Distance from mutation to the PPI interface |
| A549_p53WT_Nutlin-3_Z-score | MTR3D (5 Å window size) | -0.217 | Missense Tolerance Ratio, available on https://biosig.lab.uq.edu.au/mtr3d/ |
| A549_p53WT_Nutlin-3_Z-score | MTR3D (6 Å window size) | -0.208 | Missense Tolerance Ratio, available on https://biosig.lab.uq.edu.au/mtr3d/ |
| A549_p53WT_Nutlin-3_Z-score | mCSM-PPI1 (monomer A&B) ΔΔG | -0.205 | Effect of mutation on PPI binding computed using mCSM-PPI1 |
| A549_p53WT_Nutlin-3_Z-score | *psi* | 0.198 | Torsion angle |
| A549_p53WT_Nutlin-3_Z-score | mCSM-PPI2 (monomer A&B) ΔΔG | -0.089 | Effect of mutation on PPI binding computed using mCSM-PPI2 |
| A549_p53WT_Nutlin-3_Z-score | MTR v1 (41 codon window size) | 0.066 | Missense Tolerance Ratio, available on https://biosig.lab.uq.edu.au/mtr-viewer/ |
| A549_p53WT_Nutlin-3_Z-score | ENCoM ΔΔG | -0.063 | Effect of mutation on protein stability computed using ENCoM |
| A549_p53WT_Nutlin-3_Z-score | Δvibrational entropy | 0.063 | Change of vibrational entropy generated using ENCoM |
| A549_p53WT_Nutlin-3_Z-score | MTR v1 (21 codon window size) | 0.057 | Missense Tolerance Ratio, available on https://biosig.lab.uq.edu.au/mtr-viewer/ |
| A549_p53WT_Nutlin-3_Z-score | MTR v1 (31 codon window size) | 0.045 | Missense Tolerance Ratio, available on https://biosig.lab.uq.edu.au/mtr-viewer/ |
| A549_p53WT_Nutlin-3_Z-score | MTR v2 (31 codon window size) | -0.035 | Missense Tolerance Ratio, available on https://biosig.lab.uq.edu.au/mtr-viewer/ |
| A549_p53WT_Nutlin-3_Z-score | mCSM-DNA ΔΔG | -0.030 | Effect of mutation on protein-DNA binding computed using mCSM-DNA |
| A549_p53WT_Nutlin-3_Z-score | *phi* | 0.025 | Torsion angle |
| A549_p53WT_Nutlin-3_Z-score | MTR v2 (21 codon window size) | -0.023 | Missense Tolerance Ratio, available on https://biosig.lab.uq.edu.au/mtr-viewer/ |
| A549_p53WT_Nutlin-3_Z-score | MTR v2 (41 codon window size) | 0.008 | Missense Tolerance Ratio, available on https://biosig.lab.uq.edu.au/mtr-viewer/ |
| A549_p53WT_Nutlin-3_Z-score | mCSM-NA ΔΔG | 0.000 | Effect of mutation on protein-DNA binding computed using mCSM-NA |

**FIGURES**


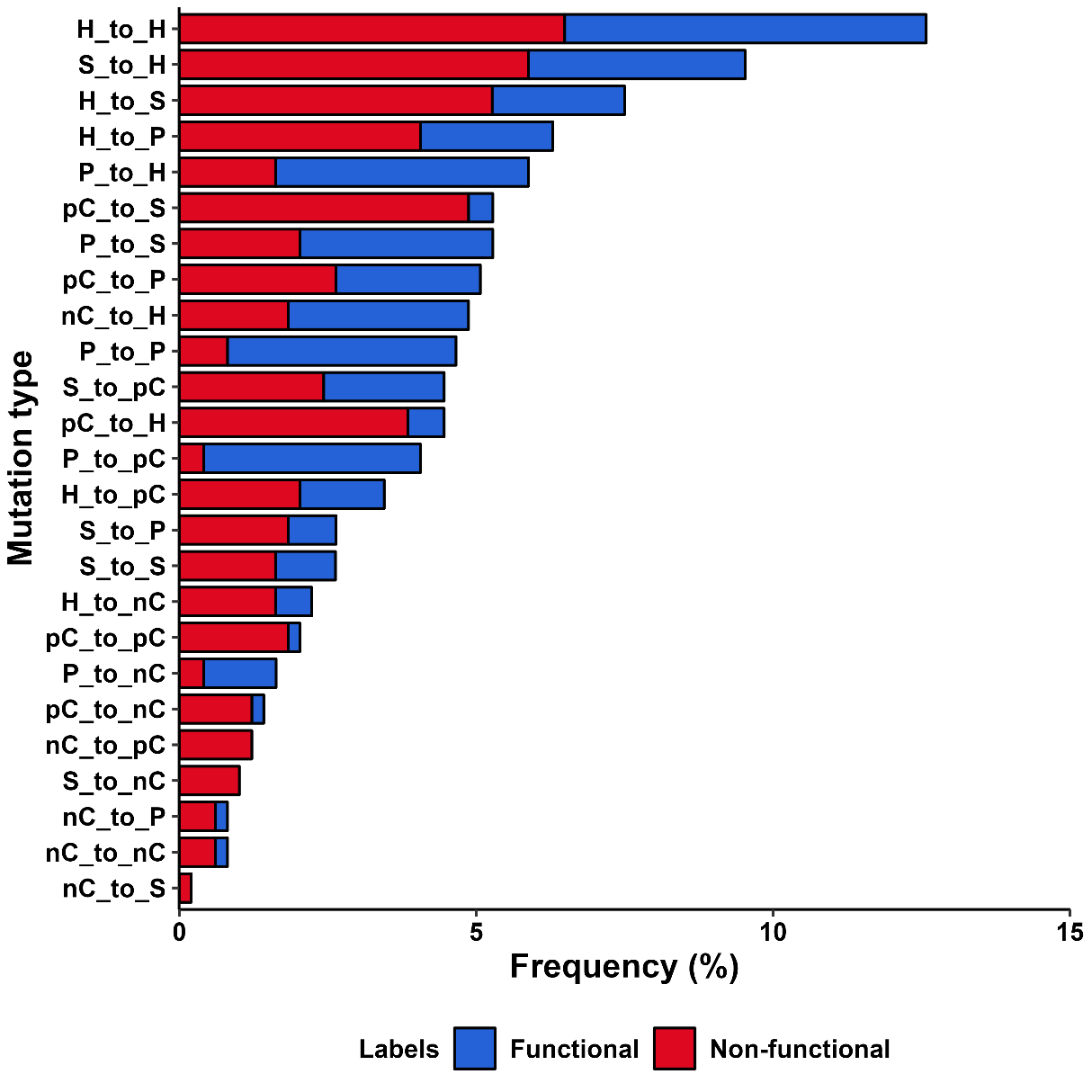


**Figure S1.** Distribution of different types of mutations in the DNA-binding domain (DBD) of p53. All 20 amino acids were assigned to one of the five groups based on their biochemical properties, including hydrophobic (A, F, I, L, M, V, W, and Y), polar (N, Q, S, and T), negative charged (D and E), positive charged (H, K, and R), and special (C, G, and P). The names of these five groups were simplified as hydrophobic (H), polar (P), negative charged (nC), positive charged (pC), and special (S).


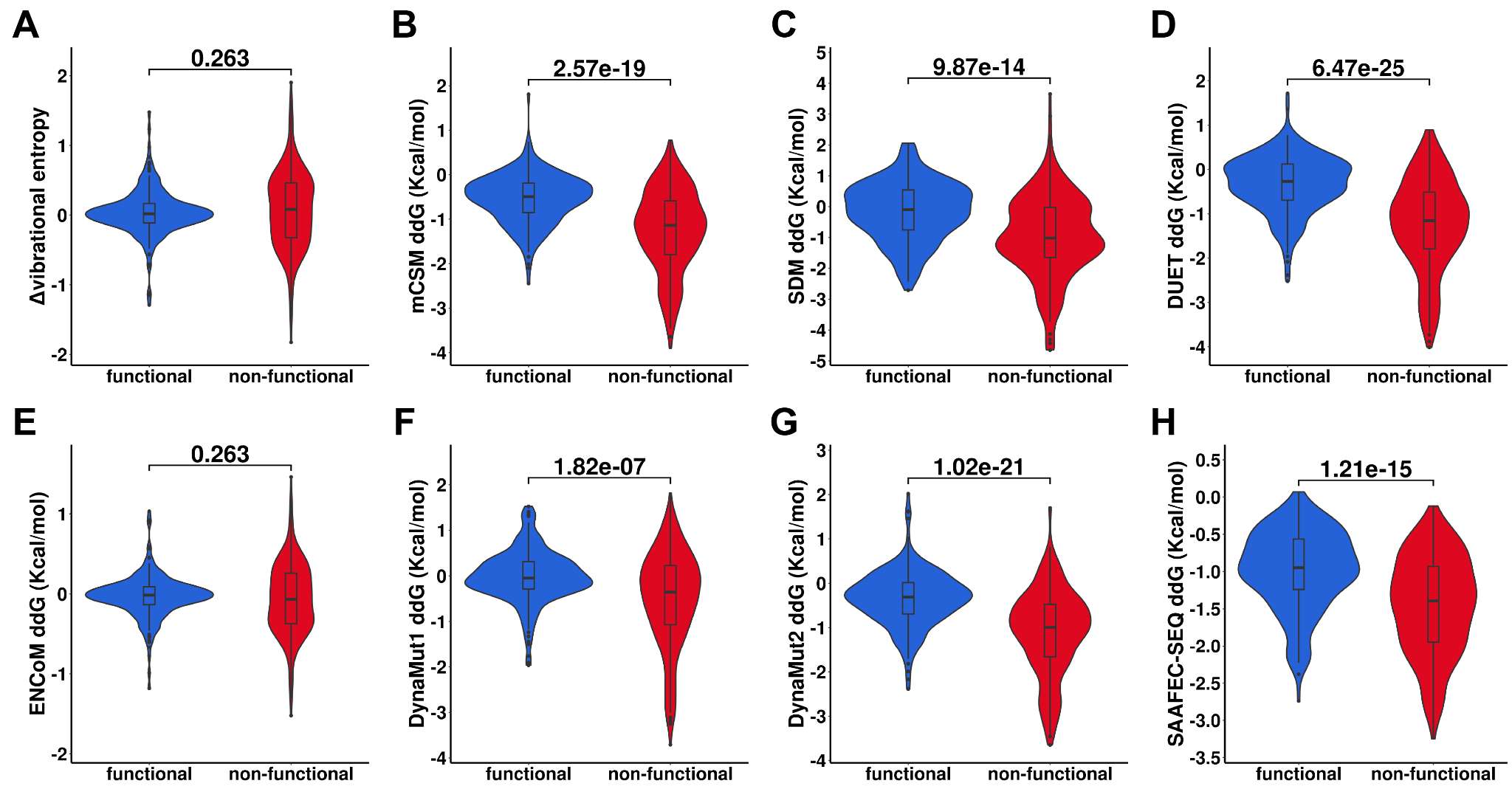


**Figure S2.** Qualitative analysis on the mutation effect of protein dynamics (A) and protein stability (B-H) between functional and non-functional mutations in the DBD of p53. Protein dynamics was computed by Normal Mode Analysis using ENCoM (A). Mutation effect of protein stability was computed by mCSM-Stability (B), SDM (C), DUET (D), ENCoM (E), DynaMut1 (F), DynaMut2 (G), and SAAFEC-SEQ (H), respectively. Wilcoxon signed-rank test was used to compare the mean of each feature at 5% significance level. Protein stability was measured using ΔΔG (Kcal/mol) with 0 as a cutoff (ΔΔG < 0: destabilising; ΔΔG > 0: stabilising).


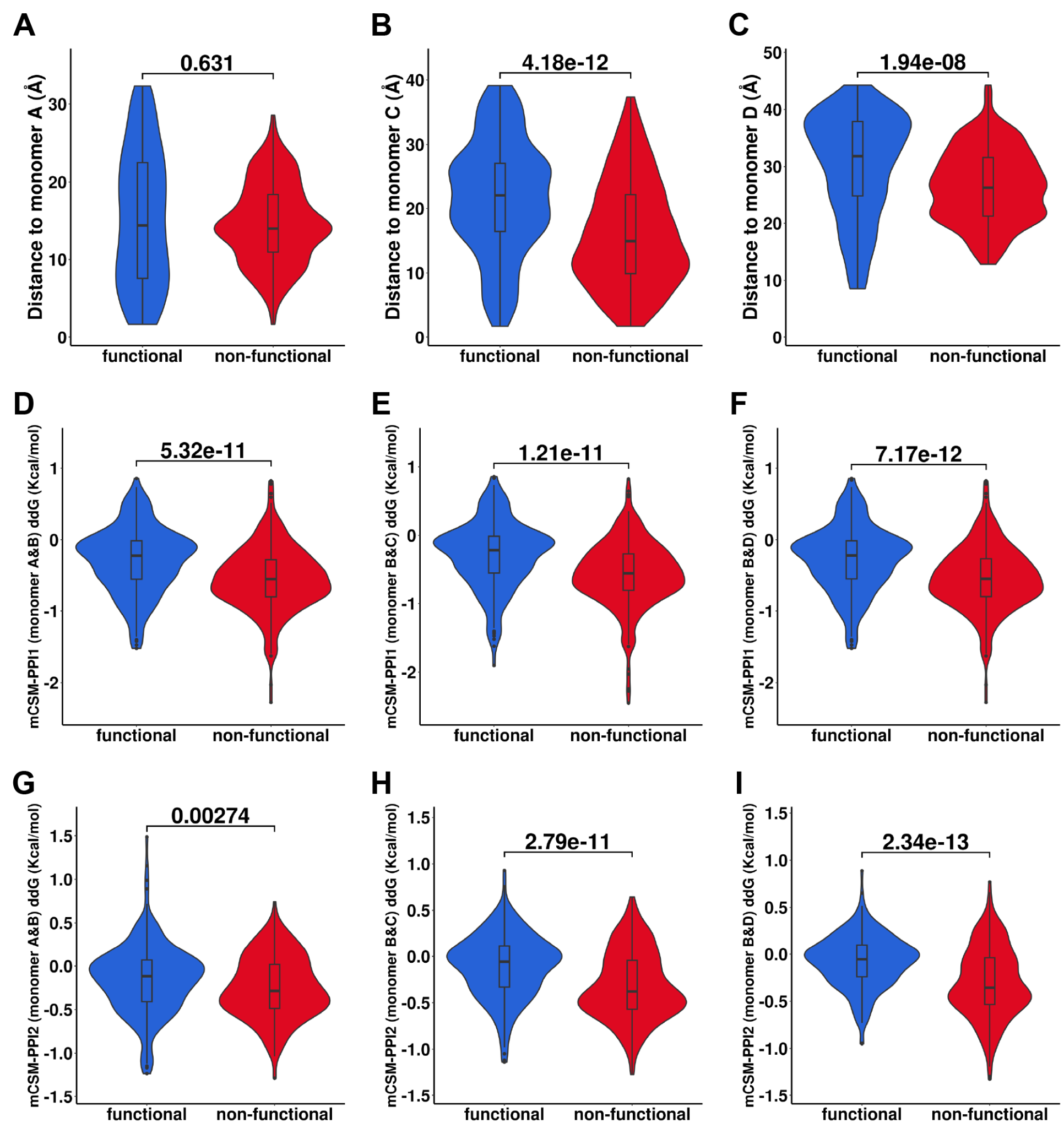


**Figure S3.** Qualitative analysis on the distance from the mutation site of monomer B to the other monomers (A-C) and the mutation effect of protein-protein binding affinity computed by mCSM-PPI1 (D-F) and mCSM-PPI2 (G-I) between monomer B and the other monomers between functional and non-functional mutations in the DBD of p53. Tetrameric p53 is formed as a dimer of dimer. Monomer A and B are at the dimer-dimer interface, while monomer B and C are at the dimer interface. Monomer B and D are at the diagonal position. Wilcoxon signed-rank test was used to compare the mean of each feature at 5% significance level. Binding affinity was measured using ΔΔG (Kcal/mol) with 0 as a cutoff (ΔΔG < 0: decreased affinity; ΔΔG > 0: increased affinity).


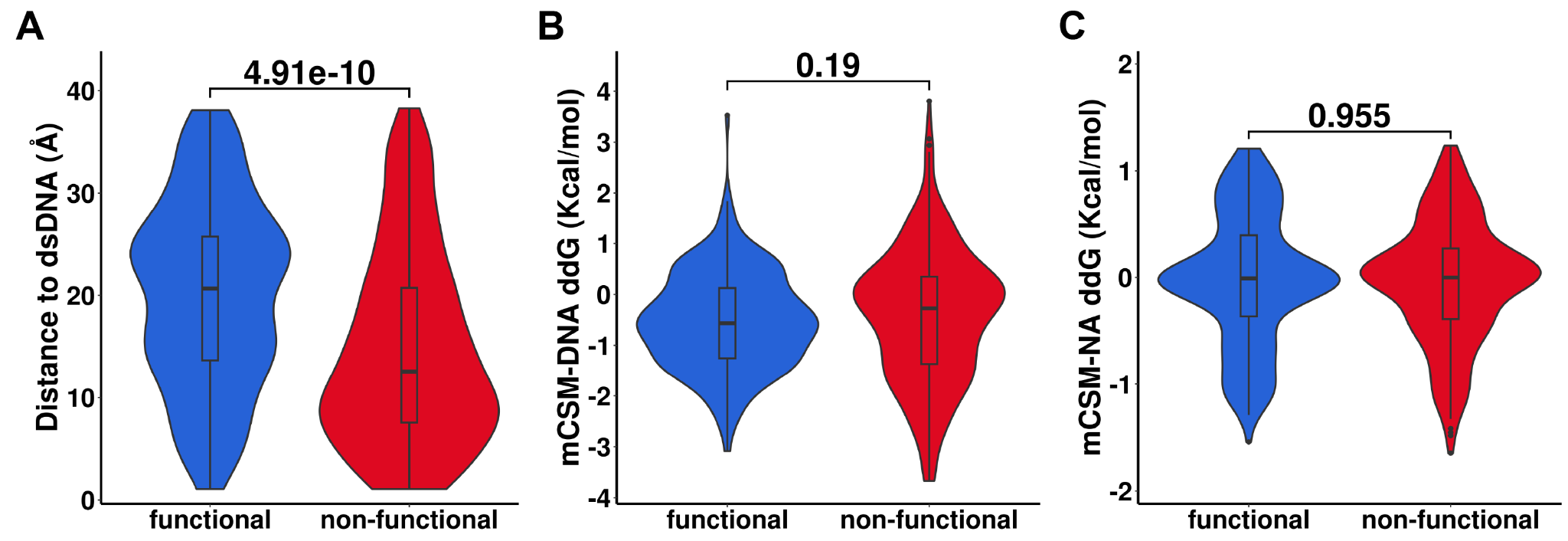


**Figure S4.** Qualitative analysis on the distance from the mutation site to the dsDNA molecule (A) and the mutation effect of protein-dsDNA binding affinity computed by mCSM-DNA (B) and mCSM-NA (C) between functional and non-functional mutations in the DBD of p53. Wilcoxon signed-rank test was used to compare the mean of each feature at 5% significance level. Binding affinity was measured using ΔΔG (Kcal/mol) with 0 as a cutoff (ΔΔG < 0: decreased affinity; ΔΔG > 0: increased affinity).


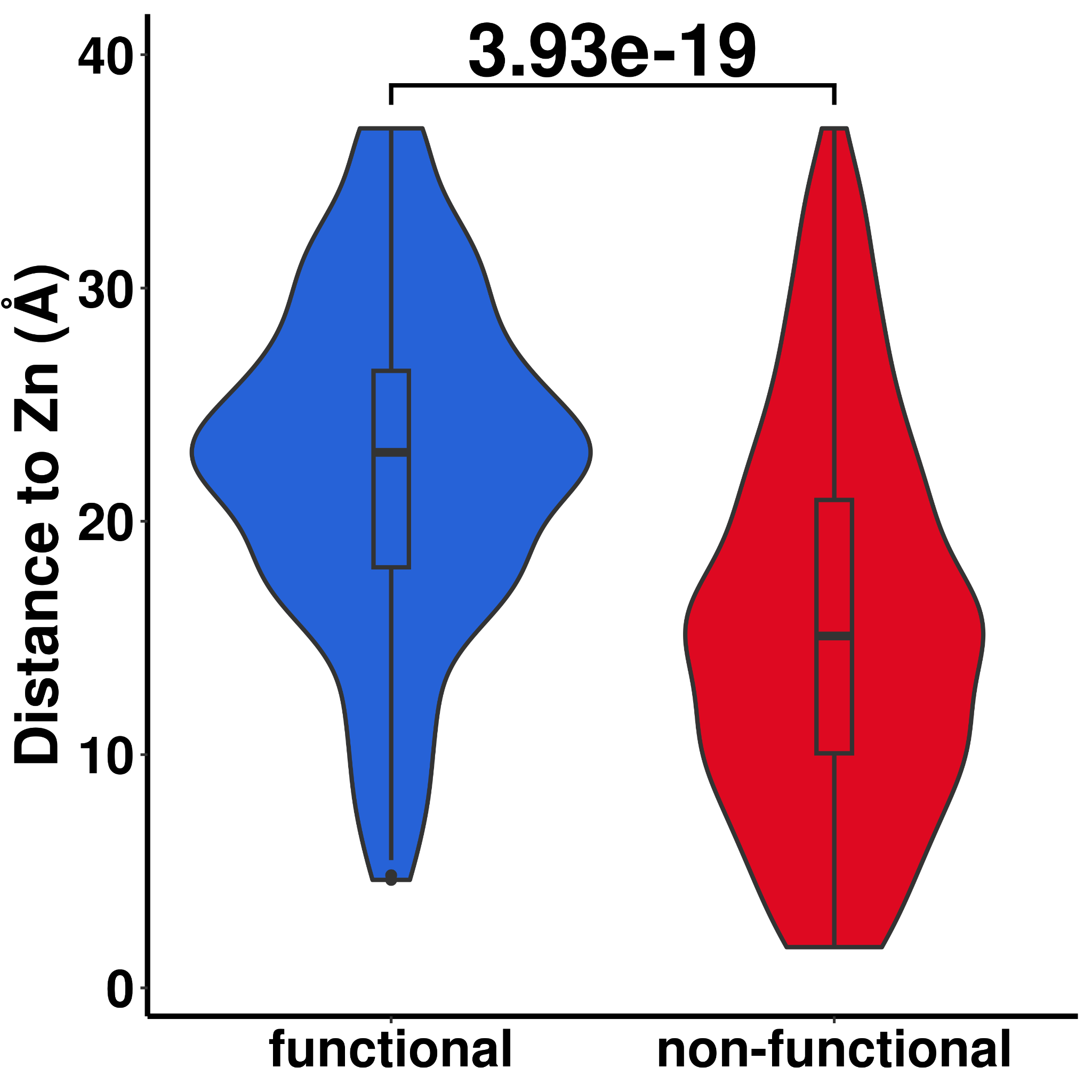


**Figure S5.** Qualitative analysis on the protein-metal interaction between functional and non-functional mutations in the DBD of p53. The distance from mutation site to the Zn^2+^ atom was used as a representative of protein-metal interaction. Wilcoxon signed-rank test was used to compare the mean of each feature at 5% significance level.


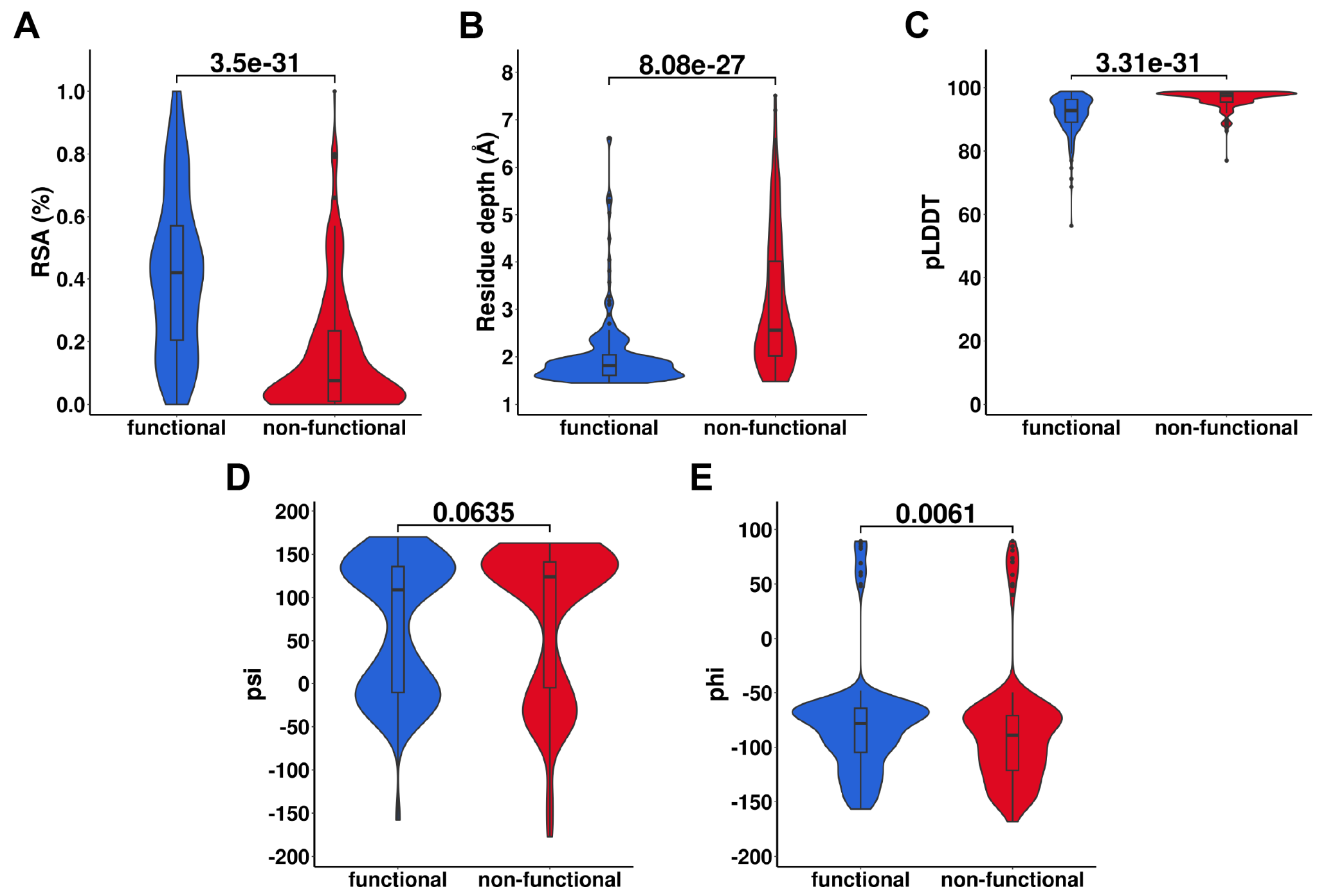


**Figure S6.** Qualitative analysis on the structural environment, including Relative Solvent Accessibility (RSA) (A), residue depth (B), protein disorderness measured by the residue confidence score of AlphaFold2 model (C), and the dihedral angles psi (D) and phi (E), between functional and non-functional mutations in the DBD of p53. Wilcoxon signed-rank test was used to compare the mean of each feature at 5% significance level.


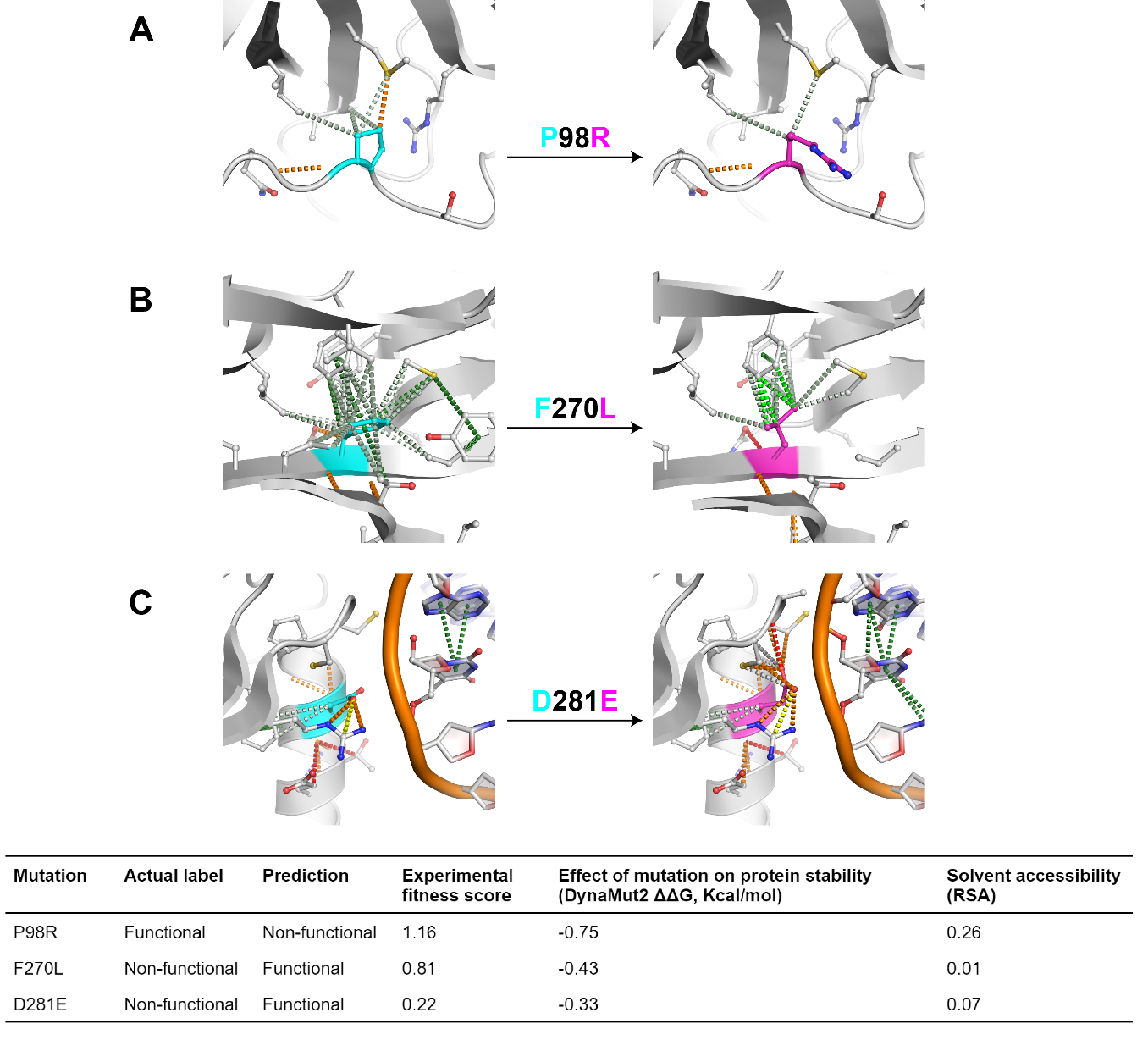


**Figure S7.** Case study of three mutations in the dataset which were wrongly classified. Here is a brief analysis of these data with irregular pattern. A reliable cutoff for classifying non-functional mutations using the fitness scores of the experimental assay was 0.81, illustrated in Table S2. According to the fitness score and a notable change on protein stability, P98R was likely to cause non-functional effect, but it actually caused mild effect to the protein function, supported by the mild change of the residue contacts (A). Similarly, the fitness score of F270L was closed to 0.81, and this mutation mildly destabilised the protein structure, which tended to be a functional mutation, but it was a non-functional mutation, supported by the mild change of the residue contacts (B). In terms of D281E, these mutations had the wild-type and mutant residues with similar biochemical properties, and the fitness score was also low. However, this mutation would cause deleterious effect to p53 function (C).


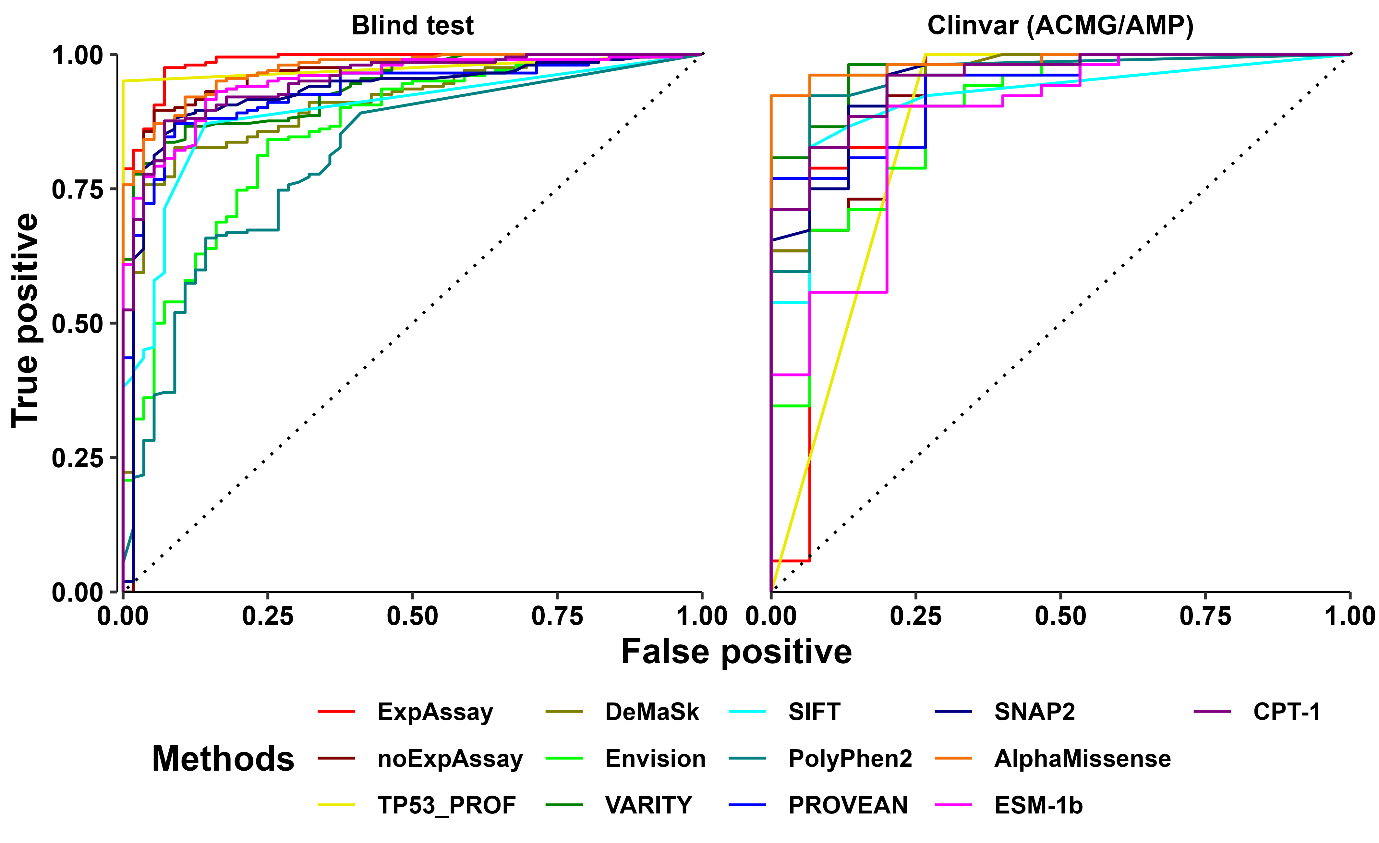


**Figure S8.** Receiver operating characteristic (ROC) curve on the blind test and the independent clinical validation of different p53 mutation effect predictors.


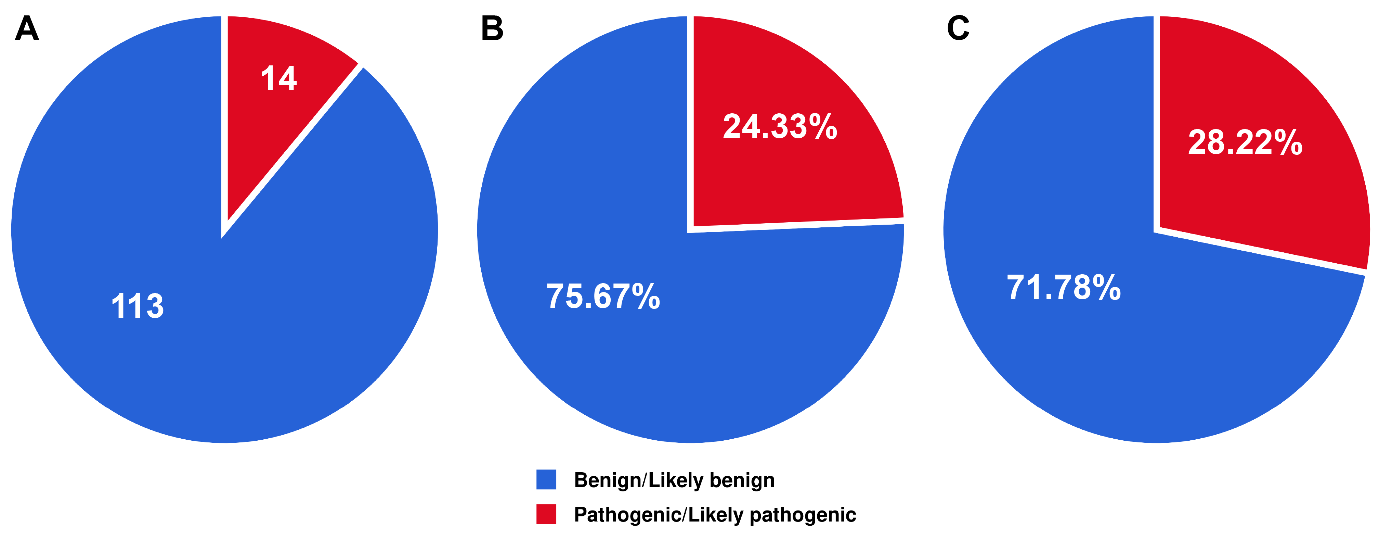
**Figure S9.** Distributions of predicted labels, including the mutations of Variant of Unknown Significance (VUS) in independent clinical validation (A) and the results of saturation mutagenesis of ExpAssay model (B) and noExpAssay model (C). Actual count of the annotated mutations was displayed for VUS mutations, while percentage was used for saturation mutagenesis (B, C).


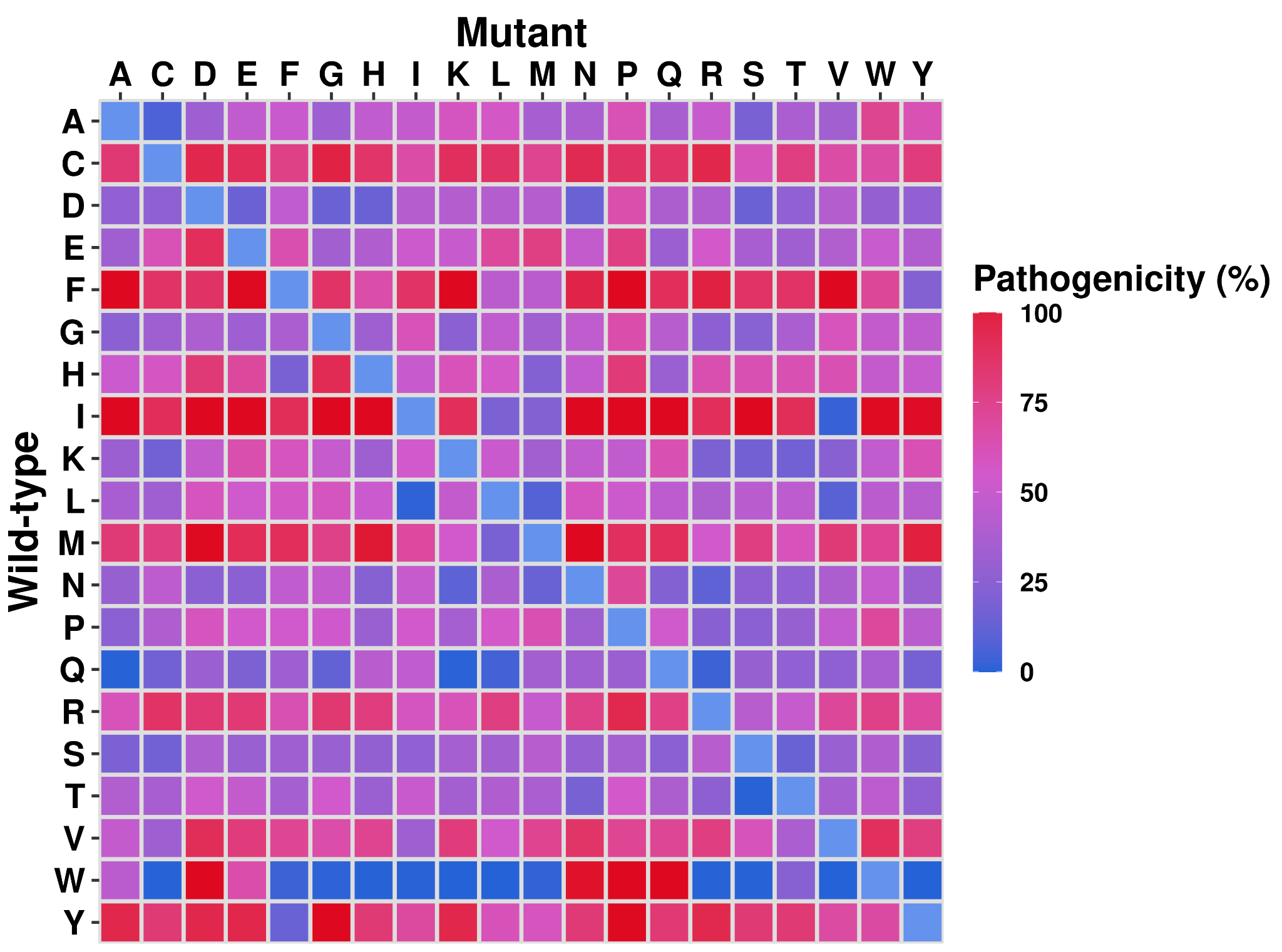


**Figure S10.** Distributions of pathogenic mutation types in DBD of p53.
